# Normalized L3-based link prediction in protein–protein interaction networks

**DOI:** 10.1186/s12859-023-05178-3

**Published:** 2023-02-22

**Authors:** Ho Yin Yuen, Jesper Jansson

**Affiliations:** 1 Department of Biomedical Engineering, The Hong Kong Polytechnic University, Hong Kong, China; 2 Graduate School of Informatics, Kyoto University, Kyoto, 606-8501 Japan

**Keywords:** Protein–Protein Interaction, Link Prediction, L3 Principle, Network Modeling, Complex Network, Graph Theory

## Abstract

**Background:**

Protein–protein interaction (PPI) data is an important type of data used in functional genomics. However, high-throughput experiments are often insufficient to complete the PPI interactome of different organisms. Computational techniques are thus used to infer missing data, with link prediction being one such approach that uses the structure of the network of PPIs known so far to identify non-edges whose addition to the network would make it more sound, according to some underlying assumptions. Recently, a new idea called the *L3 principle* introduced biological motivation into PPI link predictions, yielding predictors that are superior to general-purpose link predictors for complex networks. Interestingly, the L3 principle can be interpreted in another way, so that other signatures of PPI networks can also be characterized for PPI predictions. This alternative interpretation uncovers candidate PPIs that the current L3-based link predictors may not be able to fully capture, underutilizing the L3 principle.

**Results:**

In this article, we propose a formulation of link predictors that we call *NormalizedL3* (*L3N*) which addresses certain missing elements within L3 predictors in the perspective of network modeling. Our computational validations show that the L3N predictors are able to find missing PPIs more accurately (in terms of true positives among the predicted PPIs) than the previously proposed methods on several datasets from the literature, including BioGRID, STRING, MINT, and HuRI, at the cost of using more computation time in some of the cases. In addition, we found that L3-based link predictors (including L3N) ranked a different pool of PPIs higher than the general-purpose link predictors did. This suggests that different types of PPIs can be predicted based on different topological assumptions, and that even better PPI link predictors may be obtained in the future by improved network modeling.

**Supplementary Information:**

The online version contains supplementary material available at 10.1186/s12859-023-05178-3.

## Introduction

In the post-genomic era, high-throughput techniques are being developed to measure and analyze high-level molecular activities. This includes static assays like mRNA sequencing that can capture the differential expression of biological processes at a time point[1], or single-cell RNA sequencing to map out the progressive shift in cellular heterogeneous activities based on cell populations[2]. One of the oldest and most important contributions to the field has been the development of large-scale characterizations of protein interactions[3]. The abundance of available protein interaction datasets and their integration has in turn led to the creation of Protein–Protein Interaction (PPI) networks [4, 5], which can be used as reference *interactomes* that display the interactions between the proteins of a particular organism. A PPI network is a form of complex network in which every node represents a protein and every edge indicates that two proteins can interact with each other. Since PPIs describe signal transduction of protein physical docking[6], large-scale studies can provide insights into the molecular machinery of living systems[7]. On a basic level, researchers can abstract biological components such as signaling pathways as a chain of PPIs[8], or protein complexes as graph clusters[9] for network analysis. In larger-scale studies, PPI networks can even be used as building blocks that associate with other biological networks for better prioritization of candidate disease proteins or improved drug repurposing[10, 11].

The basis of meaningful and comprehensive discoveries is a complete and reliable PPI network. However, measurement errors or incomplete experimental data may lead to some parts of the constructed PPI network having the wrong structure. For this reason, computational tools have been developed to evaluate the accuracy of the proposed edges in an existing PPI network or to find good candidates for new edges whose addition to the network would make it more biologically sound. The most direct approaches use protein sequences data[12, 13], since protein sequences compare proteins’ functions genetically. Some of the other approaches include the use of protein structures, RNA co-expression, and protein annotations[14][15]. Undoubtedly, the success of these methods stems from utilizing features to describe proteins, subsequently characterizing PPIs. A recent survey of existing approaches to PPI predictions can be found in [16].

On the other hand, general-purpose link prediction techniques have been developed for complex networks such as computer networks, recommender systems, and social networks[17]. These link predictors can also be applied to PPI data, but they are usually not specific enough to characterize PPIs well and there are no guarantees on their correctness and reliability. Due to this concern, Kovács et al.[18] introduced a novel link predictor based on a biological motivation that they called the *L3 principle*. It relies on the principle that, from a structural perspective, two proteins that share many common neighbors are likely to have a similar interaction interface. Consequently, two proteins linked by many different paths of length three will have a higher likelihood of also interacting directly with each other. Observe that this contrasts with the general network principle known as the triadic closure principle[19] or Common Neighbor[20], which typically assumes that the number of paths of length two between two nodes is correlated to the likelihood of them also being directly connected by a single edge. Assuming that the L3 principle holds, the *L3* link predictor infers new PPIs by assigning a score to every pair of non-adjacent nodes based on information about paths of length three between them, and letting those node pairs with the highest scores be the candidate PPIs. The study by Kovács et al.[18] also showed computationally that the *L3* link predictor outperforms a vast number of general link predictors, including Common Neighbor. Since then, various studies have already successfully improved existing network biology techniques by incorporating the L3 principle, including drugs-disease network analysis[21] and protein fold recognition[22].

Despite the strength of the L3 principle, some researchers claim that our understanding of the L3 link predictor is limited and that it was derived empirically rather than from any theoretical knowledge[23]. Indeed, it would be more appropriate to regard the L3 link predictor as an *approximation* since its normalization term (the use of a square root) was derived empirically. While normalization is generally a part of any link predictor and each one has a different way of addressing it, the definitions are often supported by their corresponding biological motivations, which is not the case in the normalization in the L3 predictor.

An alternative approach would be to evaluate the fitness to the L3 principle by characterizing and modeling neighborhoods of PPIs more precisely, namely to reward desirable graph structures such as paths of length three, and penalize undesirable graph structures such as paths of length two. In this article, we define the link predictor in a way that more accurately corresponds to the biological motivation behind the L3 principle. Our method is named *NormalizedL3* (*L3N*). We demonstrate computationally that L3N is better at inferring unknown PPIs than the previous methods, which gives further evidence that the structure of PPI networks can be accurately reconstructed from partial data by using link predictions.

We would like to remark here that the preliminary conference version of this article[24] contains an error in the presented formula for $P_{xy}^{(L3E)}$ (Formula (4) in [24]) and that the experimental results were obtained using a slightly different (and correct) version of the formula that was implemented as intended in the program code that was provided. In this article, we have corrected the error and also generalized the formula to further improve the performance of our link predictor; see Formulas (6) and (7) in Sect. 4.2 below.

The article is organized as follows. Section 2 reviews some known general and PPI-specific link prediction techniques. Then, we provide the problem definition and the formulation of L3N in Sect. 3 and Sect. 4, respectively. Using the materials described in Sect. 5, we evaluate the performance of the link predictors on simulated datasets of a particular structure in Sect. 6. In Sect. 7, we then evaluate the predictive power and biological significance of L3N using statistical metrics, and present some observations on the differences between L3N and other link predictors. Finally, in Sect. 8, we summarize our findings and discuss potential future improvements of PPI link prediction.

## Previous work

Link prediction infers new edges based on the properties of the nodes as well as the overall topology of the existing edges[17]. Many classes of link prediction approaches exist, and this article will focus on *similarity-based* link predictions, where candidate edges are selected based on the similarity of nodes’ immediate or extended neighborhoods by iterating every pair of nodes. Some link predictors of this type are reviewed next. From here on, for any node *a*, let *N*(*a*) denote the set of neighbor nodes of *a*, and for any set *A* of nodes, let $N(A) = \bigcup _{a\in A} N(a)$. There are also many different general link prediction approaches that model the problem beyond iterating candidate edges, for example it has been modified as a random walk problem[25], a linear optimization problem[26], and more. For a more comprehensive review, see [17].

### General link prediction

The Common Neighbors (CN) concept originates from social networks[20]. It models a social phenomenon: the more friends two individuals share, the more likely they are to also be friends of each other. Then, the CN score of any two nodes *a* and *b* is $|N(a)\cap N(b)|$. The assumption here is that the higher the CN score, the more confident we can be that the two nodes should be adjacent. In the context of PPIs, a high CN score of two proteins implies that they have similar functions[27]. That is, if two proteins interact with a similar set of proteins then their functions should be similar.

However, a high-degree node will contribute to the CN scores of many more node pairs than a low-degree node will. To reduce the influence that a single node may have, one can penalize high-degree nodes in some way. To do so, the Resource Allocation (RA) algorithm[28] makes high-degree nodes contribute less by using the following formula instead for every pair of nodes *a* and *b*: $\sum _{z\in N(a)\cap N(b)} \frac{1}{|N(z)|}$. In addition to RA, there exist many other normalization schemes. In the Adamic-Adar (AA) Index[29], a logarithmic modifier (whose motivation comes from social networks mining) is used to do the normalization: $\sum _{z\in N(a)\cap N(b)} \frac{1}{\log (|N(z)|)}$. For a survey of the normalization schemes used in many other general link predictors, see[17].

### PPI-specific link prediction

Link predictors can also consider parts of the network beyond the immediate neighborhoods of nodes. For example, in the context of PPI networks, [30] applies random walks to identify and connect pairs of nodes with similar distances to the other nodes in the network.

In another study, Nakajima et al.[31] used protein complex datasets on top of PPI datasets to investigate how many PPIs might be missing from those PPI datasets. Assuming that each protein complex must induce a connected subgraph in the corresponding PPI network, the minimum number of edges that have to be added to ensure that this condition holds in the network thus gave lower bounds on the number of missing PPIs in various databases. This also shows how PPI datasets can be augmented with external feature data, utilizing the biological context.

**Fig. 1:**
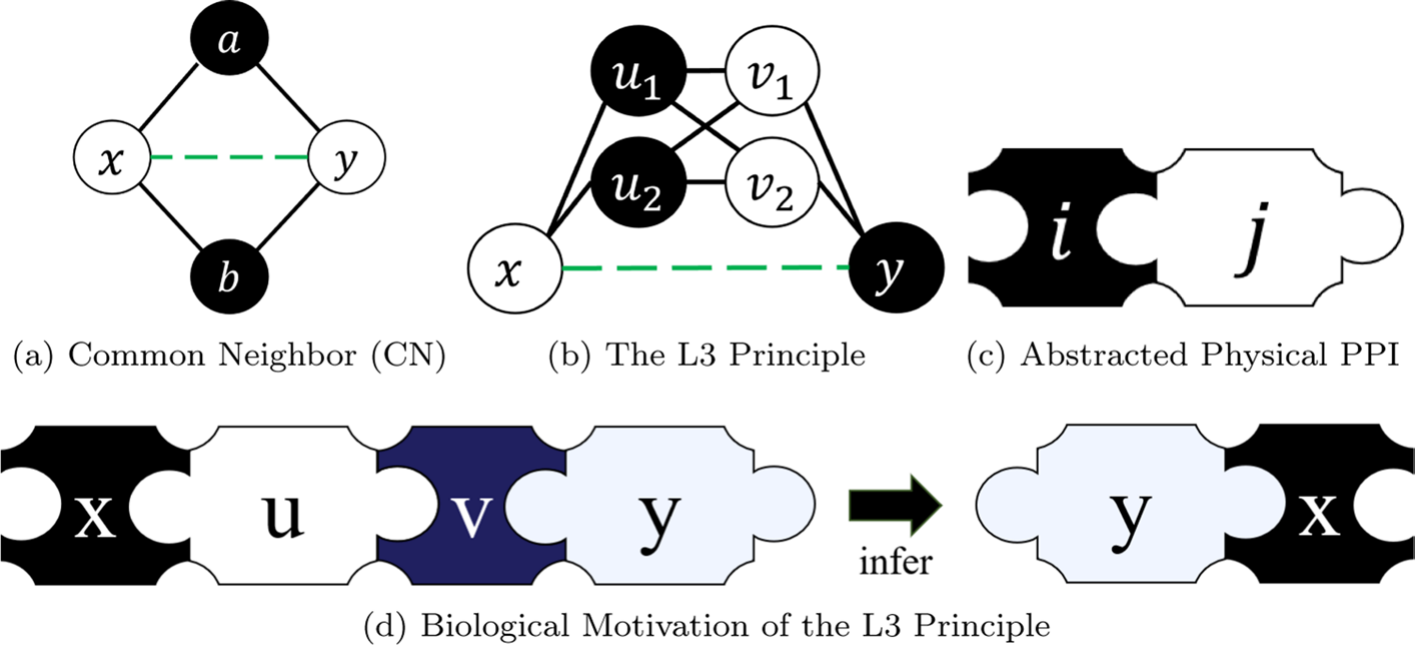
Principles behind link prediction in PPI networks. (**a**) and (**b**) illustrate conditions that would lead CN and the L3 principle, respectively, to predict that an edge between the two non-adjacent nodes *x* and *y* is in fact missing. (**c**) A graphical representation of the occurrence of a physical PPI between protein *x* and protein *y*. (**d**) Using the abstraction in (**c**), if the PPIs are arranged as shown on the left, we can infer the existence of a PPI between protein *y* and protein *x* as shown on the right

Finally, in the study of our focus[18], Kovács et al. presented the so-called *L3 algorithm*, which is biologically motivated by the following observation: Since a physical PPI is the physical docking of two proteins, it can only occur if the interfaces of the two proteins are compatible. Now, if nodes *x* and *y* in a PPI network share many neighbors, it can be expected that the interface of *x* is similar to the interface of *y*. Two proteins with identical or nearly identical interfaces are usually not compatible (they cannot dock with each other), which means that the PPI network will not have an edge between *x* and *y* in this case. See Fig. 1a for an illustration. On the other hand, if there are many paths of length 3 between *x* and *y* in the network then *x* and *y* are likely to be compatible, as shown in Fig. 1b. Following standard graph theory notation, $P_{3}$ will denote an undirected length-2 path consisting of three nodes and two edges, and $P_{4}$ will denote an undirected length-3 path consisting of four nodes and three edges. Using this notation, the observation above can be stated as: the more $P_{4}$-subgraphs and the fewer $P_{3}$-subgraphs that connect a pair of nodes *x* and *y*, the more certain it is that *x* and *y* should be connected by an edge. From here on, we shall refer to this principle as the *L3 principle*. Note that the actual formulation of the L3 link predictor also involves a normalization term that will be explained in the next section.

After the L3 principle was proposed[18], other researchers have taken inspiration from it to formulate new link predictors for PPI networks. This includes *CH2_L3* (abbreviated as *CH2* below)[23], a link predictor that extends the general link predictor *CRA*[32], as well as the *Sim*[33] link predictor. Both of these are similarity-based link predictions that use information from L3 neighborhoods, just like our method L3N. For this reason, they are also included in the experimental comparison below. The mechanisms of CH2 and Sim are described in more detail in Sect. 4.1 and Sect. 5.2.

## Preliminaries

### Problem definitions

Given an undirected graph $G=(V,E)$, the task is to determine, for each pair of non-adjacent nodes in *V*, whether or not an edge between them should be added to *E*. Every non-adjacent node pair $\{x,y\}$ will be assigned a score $P_{xy}$ that measures, in a relative sense, the confidence with which one can say that *x* and *y* should be connected by an edge. In other words, among all non-adjacent node pairs, the pair $\{x,y\}$ will be considered to be the best PPI candidate if $P_{xy}$ is larger than $P_{x'y'}$ for all other node pairs $\{x',y'\}$. As mentioned in Sect. 2.2, predictions assuming the L3 principle require counting the number of $P_{4}$-subgraphs between pairs of nodes. For this purpose, define $U = N(x)\cap N(N(y))$ and $V = N(y)\cap N(N(x))$, i.e., let *U* be the set of neighbors of *x* at distance 2 from *y* and analogously for *V*. Then, every $P_{4}$-subgraph between *x* and *y* is an undirected simple path of the form (*x*, *u*, *v*, *y*), where $u\in U$ and $v\in V$. Note that a node may belong to *N*(*x*) as well as *N*(*y*) and also to both *U* and *V*, in which case it will be able to take the role of either *u* or *v* in a $P_{4}$-subgraph. With these definitions, one can count the number of $P_{4}$-subgraphs between *x* and *y* using Formula (1). This kind of double summation will be abbreviated as in Formula (2) to simplify the notation from now on.

1 $$\begin{aligned}{} & {} P_{xy}^{(1)} = \sum _{i=1}^{|U|} \sum _{j=1}^{|V|} {\left\{ \begin{array}{ll} 1 &{} if u_{i} \in N(v_{j})\\ 0 &{} otherwise \end{array}\right. } \end{aligned}$$

2 $$\begin{aligned}{} & {} P_{xy}^{(1)} = \sum _{U,V} 1 \end{aligned}$$

In a link predictor, normalization is usually applied to compensate for possible issues introduced by relying on a particular principle. E.g., in the case of L3-based link predictors, since high-degree nodes in the sets *U* and *V* will contribute to many more $P_{4}$-subgraphs than low-degree nodes, this may give them a disproportionate influence on the value of $P_{xy}^{(1)}$. Hence, Formula (2) could be adjusted to penalize high-degree nodes. The L3 link predictor [18] does this by using a square root modifier according to Formula (3) below.

3 $$\begin{aligned} P_{xy}^{(L3)} = \sum _{U,V} \frac{1}{\sqrt{|N(u_{i})|\cdot |N(v_{j})|}} \end{aligned}$$

### Our contributions

We observe that the normalization modifier in Formula (3) does not completely implement the L3 principle. More precisely, Formula (3) only uses the set *U*, the set *V*, and the node degrees to evaluate an *xy*-node pair. Not only does it not take $P_{3}$-subgraphs into account, it may also give an undeservedly low score to some good *xy*-node pair simply because its intermediate *u*- and *v*-nodes happen to have high degrees. *L3N* addresses these problems by employing an alternative approach to normalization by network modeling. Instead of attributing the weighting of each $P_{4}$ to the square root term as in Formula (3), we consider whether the neighborhood of each *xy*-node pair is ideal under the L3 principle, i.e. compared to a model network that fully follows L3 principle. For example, in a $P_{4}$ consisting of four nodes (*x*, *u*, *v*, *y*), a high-degree node *v* could provide strong evidence for the existence of an *xy*-link if $N(v) = N(x)$, since this implies that *x* and *v* are structurally similar, and therefore *x* is structurally compatible with *y* (and symmetrically, *v* is compatible with *y*). By exhaustively considering all the possible conditions like the one above that we gave as an example, we can evaluate how strongly the $P_{4}$s between a candidate PPI follow the L3 principle. These evaluations also prevent high-degree nodes being undeservedly normalized, since our formulations directly compare the relative (and hence normalized) difference between neighborhoods comprehensively. This yields a normalized L3 predictor that we call L3N.

**Fig. 2:**
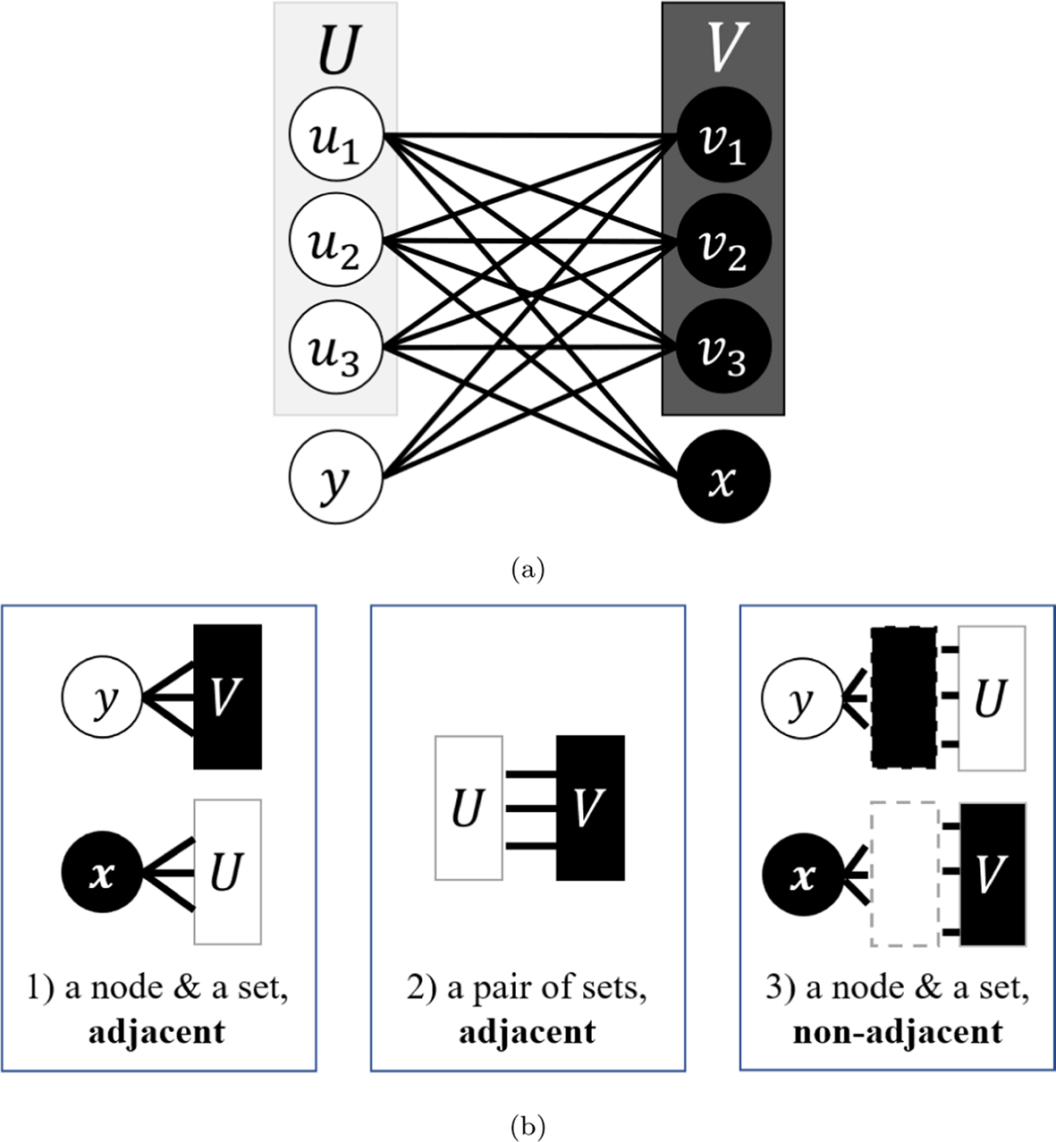
By modeling an ideal L3 graph, we illustrate the conditions using that would lead the L3 principle to assign the maximum possible score to $P_{xy}$ (relative to $P_{xy}$ obtained in non-ideal L3 graphs). **a** An example of an ideal L3 graph with the four L3-elements *x*, *U*, *V*, and *y*. **b** Three measures of how well a (possible non-ideal) L3 graph fits the L3 principle based on its L3-elements

## Detailed formulation of L3N

To describe the properties that characterize the L3 principle, we define an *ideal L3 graph* as a model graph that can be obtained by taking a complete bipartite graph with two parts *U* and *V*, and attaching a new node *x* as a neighbor of all nodes in *U* and attaching a new node *y* as neighbor of all nodes in *V*. This results in a graph with the four basic *L3-elements*: node *x*, node *y*, set *U*, and set *V*, which are the fundamental components of an ideal L3 graph. Figure 2a illustrates an example ideal L3 graph. Its nodes have been colored white and black in such a way that no pair of nodes with the same color are adjacent and every pair of nodes with different colors (except *x* and *y*) are adjacent. Then, to model real PPI networks, we need to consider *non-ideal L3 graphs* that can deviate from ideal L3 graphs in the following ways:

- An edge between *x* and *U* is missing, or an edge between *y* and *V* is missing.
- An edge between *U* and *V* is missing.
- An edge between two nodes in *U*, or between two nodes in *V*, exists.
- An edge between *x* and *V* exists, or an edge between *y* and *U* exists.

**Fig. 3:**
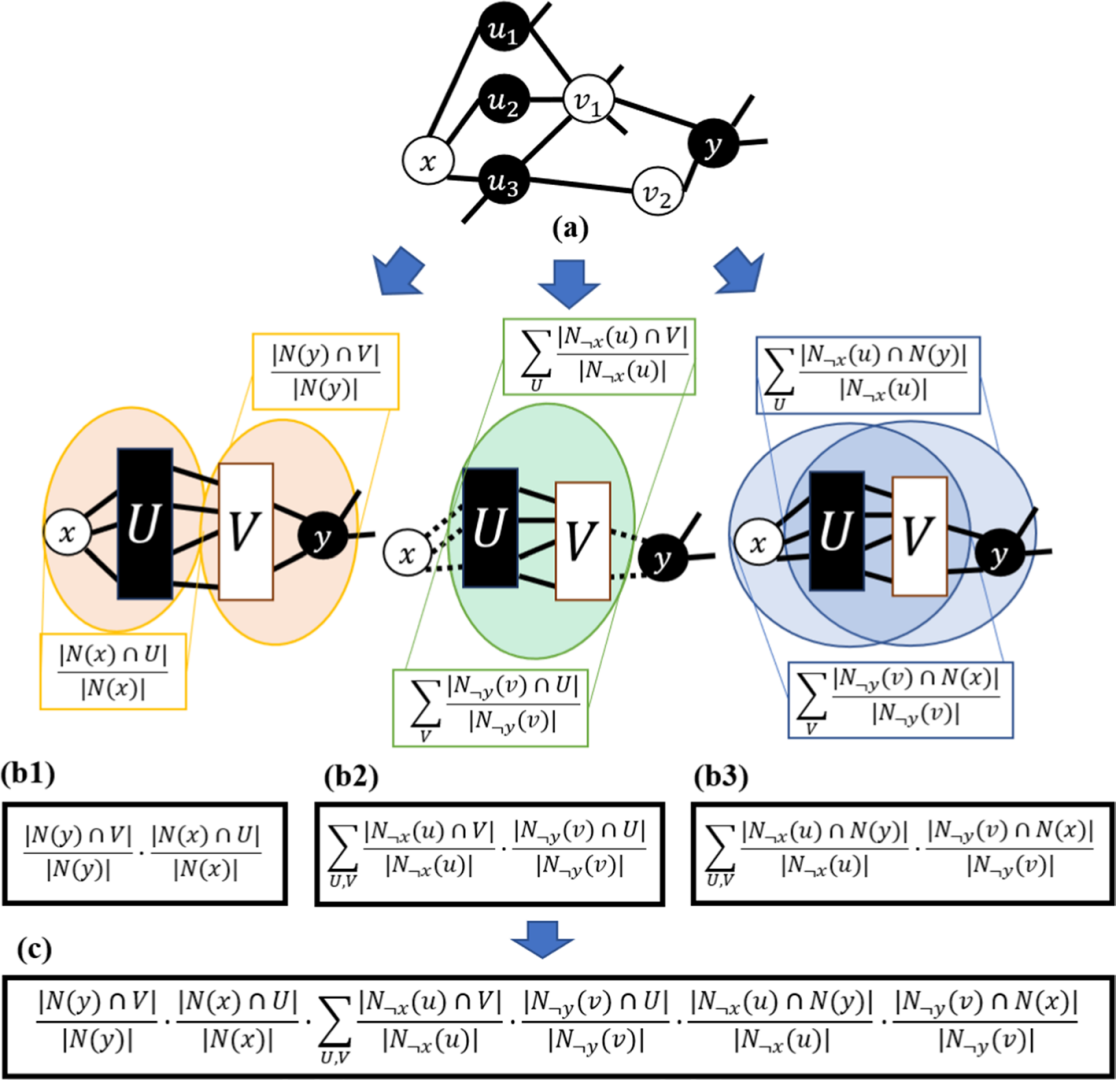
The idea behind Formula (6). **a** An L3 graph with L3-elements *x*, *U*, *V*, and *y*. **b1**–**b3** Each of the six parts of Formula (6) corresponds to one of the six conditions used to measure how close the graph is to being an ideal L3 graph. Here, the Simple Ratio $f_{1}$ from Sect. 4.1.1 has been selected as the similarity metric. **c** Combining all six parts yields Formula (6) for the score $P_{xy}^{L3N(f_{1})}$

Recall that we defined $U = N(x)\cap N(N(y))$ and $V = N(y)\cap N(N(x))$ in Sect. 3. These definitions induce, for any specified pair of nodes *x* and *y*, the L3-elements of an L3 graph whose fitness to the L3 principle can be evaluated by measuring how well the following conditions are met:

I $N(x) = U$ and $N(y) = V$ (see Fig. 2b-1)
II $N(v)\setminus \{y\} = U$ for every $v\in V$ and $N(u)\setminus \{x\} = V$ for every $u\in U$ (see Fig. 2b-2)
III $N(x) = N(v)\setminus \{y\}$ for every $v\in V$ and $N(y) = N(u)\setminus \{x\}$ for every $u\in U$ (see Fig. 2b-3)

As an example, consider a non-ideal L3 graph obtained by inserting a single edge of the form $\{u_{i}, u_{j}\}$ into an ideal L3 graph. Then, $N(u_{i})\setminus \{x\} \ne V$ and $N(u_{j}){\setminus } \{x\} \ne V$ hold, thus violating condition II. Also, $N(y) \ne N(u_{i}) {\setminus } \{x\}$ and $N(y) \ne N(u_{j}) {\setminus } \{x\}$, which violates condition III. However, this graph is still quite close to being an ideal L3 graph, so we would like our link predictor to assign a relatively high score when measuring how well conditions I, II, and III are met. Moreover, due to the incomplete nature of PPI networks, our link predictor should also be robust against false negatives that could incorrectly suggest the existence of an L3 neighborhood. Next, we review two so-called similarity metrics that will be used in the formal definition of our new link predictor.

### Similarity metrics

*Similarity metrics* are formulas that score the similarity of two sets with appropriate penalization so that the size of the two sets has a minimum effect on the score. In the case of PPI networks, the sets would be node subsets such as the neighborhood of a node. Such metrics allow us to formalize the relationships in Fig. 2b as mentioned above. In the following sections, we review two well-studied similarity metrics that will be included in our improved link predictor. (See the summary in Table 2 in Sect. 5.2 for their precise formulas.)

#### Simple ratio

Given two sets *A* and *B*, one of the simplest possible metrics is the *Simple Ratio* in Formula (4), which measures the size of the intersection relative to the size of one of the sets.

4 $$\begin{aligned} f_{1}(A,B) = \frac{|A\cap B|}{|A|} \end{aligned}$$

To give an example, the *CRA* link predictor[32] utilizes this to extend the CN principle for general link prediction (including PPI networks). CRA computes the link prediction score of node *x* and *y* by first extracting the common neighbors, $A = N(x)\cap N(y)$. Then, each node $a\in A$ is evaluated according to $f_{1}(N(a), A)$. The sum of these scores, which is $\sum _{a\in A} f_{1}(N(a),A)$, will then be the link prediction score for nodes *x* and *y*. It is defined in this way because CRA is only interested in if *N*(*a*) is a subset of *A*, regardless of the size of set *A*.

#### Jaccard coefficient

Formula (5) is the *Jaccard coefficient* [34] for set *A* and set *B*. Note that it uses a different denominator than the one in Sect. 4.1.1.

5 $$\begin{aligned} f_{2}(A,B) = \frac{|A\cap B|}{|A\cup B|} \end{aligned}$$

This evaluation assumes that both sets are equally important and that the maximum possible score can only be obtained when $A = B$. (In comparison, in Formula (4) in Sect. 4.1.1, the best score can be obtained even if $A\subsetneq B$ or $B\subsetneq A$.) This idea is utilized in the *Sim* link predictor[33]. To be precise, Sim independently scores the similarity of node *x* and nodes *v* using $f_{2}(N(x),N(v))$, and node *y* and nodes *u* using $f_{2}(N(y),N(u))$. The summation of these scores then become the link prediction score for the corresponding node *x* and *y*.

### Normalized L3 formulations

Using any similarity metric *f*, we can quantify how close a non-ideal L3 graph is to being ideal by accounting for conditions I, II, and III described at the beginning of this section as follows:

- Condition $$N(x) = U$$: use *f*(*N*(*x*), *U*)
- Condition $$N(y) = V$$: use *f*(*N*(*y*), *V*)
- Condition $N_{\lnot x}(u) = V$ for every $u \in U$: use $\sum _{U} f(N_{\lnot x}(u),V)$
- Condition $N_{\lnot y}(v) = U$ for every $v \in V$: use $\sum _{V} f(N_{\lnot y}(v),U)$
- Condition $N(x) = N_{\lnot y}(v)$ for every $v \in V$: use $\sum _{V} f(N(x), N_{\lnot y}(v))$
- Condition $N(y) = N_{\lnot x}(u)$ for every $u \in U$: use $\sum _{U} f(N(y), N_{\lnot x}(u))$

where the notation $N_{\lnot b}(a)$ is a shorthand for $N(a)\setminus \{b\}$. Then, we complete the formulation by combining them as in Formula (6). We define the link prediction score for any non-adjacent node pair $\{x,y\}$ as a sum taken over all pairs of nodes (*u*, *v*) for $u\in U$ and $v\in V$ since each $P_{4}$ that increases the likelihood of the edge between *x* and *y* corresponds to one such (*u*, *v*). Intuitively, the formula balances the influence of the number of $P_{4}$’s and the quality of each $P_{4}$: a high score will be obtained if there are many $P_{4}$’s between *x* and *y* and the subgraph induced by *x*, *U*, *V*, and *y* is an ideal L3 graph. Note that *f*(*N*(*x*), *U*) and *f*(*N*(*y*), *V*) can be evaluated outside of the inner sum since they do not depend on both *u* and *v* at the same time.

6 $$\begin{aligned}{} & {} P_{xy}^{(L3N(f))} = f(N(x),U) \cdot f(N(y),V) \cdot \sum _{U,V} f(N_{\lnot x}(u),V)\nonumber \\{} & {} \cdot f(N_{\lnot y}(v),U) \cdot f(N(x), N_{\lnot y}(v)) \cdot f(N(y), N_{\lnot x}(u)) \end{aligned}$$

Figure 3 gives a graphical explanation of Formula (6) using the similarity metric $f_{1}$ from Sect. 4.1.1. From now on, the link predictor obtained by letting $f = f_1$ in Formula (6) will be denoted by L3N($f_{1}$); similarly, plugging in $f_2$ from Sect. 4.1.2 into Formula (6) gives a link predictor that we will refer to as L3N($f_{2}$).

To illustrate the L3N formulation with an example, consider the non-ideal L3 graph mentioned previously in this section that was obtained by inserting a single edge of the form $\{u_{i}, u_{j}\}$ into an ideal L3 graph. For this graph, $u_{j}\in N(u_{i})$ although $u_{j}\notin V$, which means that $N_{\lnot x}(u_{i})$ and *V* are not completely identical and the third term in Formula (6) will be slightly smaller than its maximum possible value. Moreover, the fact that $N_{\lnot x}(u_{j}) \ne V$ will also contribute to the third term not being maximized, and $N(y)\ne N_{\lnot x}(u_{i})$ and $N(y) \ne N_{\lnot x}(u_{j})$ will prevent the sixth term from being maximized.

As another example, consider what happens if a single edge of the form $\{u_{i},y\}$ is inserted into an ideal L3 graph, thus creating a $P_{3}$ $(x,u_{i},y)$. By definition, $u_{i}\in U$ and $u_{i}\notin V$ still hold. However, *N*(*y*) will no longer be equal to *V* since *N*(*y*) now also includes $u_{i}$, $N_{\lnot x}(u_{i})$ will not be equal to *V*, and *N*(*y*) will not be equal to $N_{\lnot x}(u_{i})$, which prevents the second, third, and sixth terms in Formula (6) from achieving their maximum possible values.

Formula (6) uses neighborhoods with the node *x* or *y* excluded (e.g., $N_{\lnot x}(u)$). For normalization purposes, it may in fact be advantageous to include *x* or *y* in the neighborhoods. For example, suppose that we are evaluating (*x*, *y*) and that $N(u_i) = V_i\cup \{x\}$ and $N(u_j) = V_j\cup \{x\}$ for two nodes $u_i, u_j \in U$ and $V_i, V_j\subseteq V$ with $|V_i|\; < |V_j|$. Then $f(N_{\lnot x}(u_i), V_i) = f(N_{\lnot x}(u_j), V_j)$ is possible although it would be better to have $f(N_{\lnot x}(u_{i}), V_{i}) < f(N_{\lnot x}(u_j), V_j)$ because the larger size of $V_{j}$ provides stronger evidence that $u_j$ and $V_j$ are compatible. Here, if we use neighborhoods that include *x* then we would get $f(N(u_i), V_i) < f(N(u_j), V_j)$, which gives an offset of one (since having just the node *x* in *N*(*u*) is what prevents the two terms from being equal) and might be preferable. In fact, similar modifications also appear in other link predictors. For instance, in the CH2 predictors[23], there are also numerical offsets of one added to denominator of fractions to keep the terms from being maximized. As another example, CRA[32] includes a unit offset in the denominators of fractions, again for the purpose of keeping them from being maximized.

Formula (7) below introduces an alternative L3N formulation based on this observation, which we shall refer to as L3N’ in the evaluations in later sections. In particular, L3N’($f_1$) and L3N’($f_2$) will refer to the link predictors obtained by selecting $f = f_1$ and $f = f_2$, respectively, in Formula (7).

7 $$\begin{aligned}{} & {} P_{xy}^{(L3N'(f))} = f(N(x),U) \cdot f(N(y),V) \cdot \sum _{U,V} f(N(u),V)\nonumber \\{} & {} \cdot f(N(v),U) \cdot f(N(x), N(v)) \cdot f(N(y), N(u)) \end{aligned}$$

### Time complexity

Let *n* denote the number of nodes in *G*, *m* the number of edges in *G*, and *k* the average degree of the nodes in *G*. Also, let $\langle k^{2} \rangle$ and $\langle k^{3} \rangle$ be the average of the squares of the node degrees and the average of the cubes of the node degrees, respectively. According to previous studies, the CN link predictor can be implemented to run in $O(n \langle k^{2} \rangle )$ time [35, 36], and the L3 link predictor in $O(n \langle k^{3} \rangle )$ time [36].

To analyze the time complexity of L3N and L3N’, we proceed as follows. First, the sets *U* and *V* for every pair $\{x,y\}$ of non-adjacent nodes are constructed by checking, for each *a* in *N*(*x*) and each *b* in *N*(*y*), if the edge $\{a,b\}$ exists in *G*; if so, *a* is included in *U* and *b* is included in *V*. This step takes $O(|N(x)| \cdot |N(y)|)$ time for each $\{x,y\}$, and summing over all $\{x,y\}$ yields a total contribution of this step to the total running time that is $\sum _{x} \sum _{y} O(|N(x)| \cdot |N(y)|) = O(\sum _{x} |N(x)| \cdot \sum _{y} |N(y)|) = O(m^2)$.

Next, either Formula (6) or Formula (7) is evaluated for each pair $\{x,y\}$ of non-adjacent nodes. For each such pair, $O(|N(x)| \cdot |N(y)|)$ factors of the form *f*(*A*, *B*) are multiplied together. As above, summing over all $\{x,y\}$-pairs shows that in total, $O(m^2)$ *f*(*A*, *B*)-values need to be computed and multiplied. To compute the individual *f*(*A*, *B*)-values efficiently, all graph neighborhoods, *U*-sets, and *V*-sets are preprocessed and stored in hash tables (one hash table for each set) using $O(m^2)$ time, after which checking if a node belongs to any set of the form *N*(*a*), *U*, or *V* takes *O*(1) time. Then, the set intersection operation $A \cap B$ can be implemented by looking up each of the elements of the smaller set in the hash table for the larger set, thus taking $O(\min \{|A|,|B|\})$ time. For the set union operation $A \cup B$, one has to access all elements of both sets if the intersection is empty, which takes $O(|A|+|B|)$ time. This means that for $f = f_1$ or $f = f_2$, since every *f*(*A*, *B*) applies the intersection and union operations to sets of size at most *n*, the value of any *f*(*A*, *B*) can be obtained in *O*(*n*) time.

In summary, the time complexity of L3N and L3N’ is $O(m^2 + m^2 + m^2 \cdot n) = O(m^2 n)$. Since $m = \frac{1}{2}\cdot n \cdot k$, the time complexity can also be expressed as $O(n^3 k^2)$.

**Table 1:** Overview of the PPI datasets used in the experiments

Dataset \Number of	Size (MB)	Nodes	PPIs	Cand. PPIs	SIPs
*Synthetic PPI dataset*					
Wang et al.[37]	0.547	8,272	52,922	29,816,060.1	480
*Saccharomyces cerevisiae* (Yeast)					
BioGRID[38]	316	7085	113,116	20,045,849.4	1739
STRING[39]	85.5*	4673	94,529	9,212,026.6	0
MINT[40]	38.3	4049	16,927	5,980,266.7	0
*Homo sapiens* (Human)					
BioGRID[38]	166	24,760	452,684	220,833,040.0	2900
STRING[39]	717*	15,668	308,614	88,982,499.1	12
MINT[40]	55.0	7,534	22,324	15,493,875.9	0
HuRI[41]	161	8109	51,127	21,899,033.2	0
HI-II-14[42]	0.185**	4298	13,868	5,165,263.5	518
Hein et al.[43]	0.368**	5457	28,780	10,939,287.2	1127
Lit-BM-13[42]	0.135**	5545	11,045	8,147,585.2	890
Lit-NB-13[42]	0.064**	3391	4906	2,738,996.0	518

“Cand. PPIs” refers to the mean number of candidate PPIs for its ten sampled datasets

“SIPs” refers to the number of self-interacting proteins

*Denotes the combined file size of multiple essential metadata files

**Indicates that the (pre-processed) dataset was downloaded from the repository of the study by Kovács et al.

## Materials

In this section, we will give a brief overview of the PPI datasets that were used in our computational validations and the other link predictors that were compared to L3N.

### Datasets

Our computational validations involved three types of datasets. The first one, the primary datasets, consisted of datasets based on systemically curated PPIs from the literature, namely Lit-NB-13[42], Lit-BM-13[42], BioGRID[38], STRING[39], and MINT[40]. For BioGRID, STRING, and MINT, we selected two organisms from these three datasets that represented small-scale and large-scale data, respectively: the well-studied model yeast (*Saccharomyces cerevisiae*, strain S288C), and human (*Homo sapiens*). Lit-NB-13 and Lit-BM-13 consist only of human PPIs. We included multiple primary datasets because the methodology used to obtain them and their confidence thresholds often differ[44]. For example, STRING includes predicted PPIs, unlike the other datasets. The second type of datasets we considered were reference interactome screened and generated from a single experimental source. This includes (ordered by their publication date) HI-II-14[42], Hein et al.[43], and HuRI[41]. All the PPIs in the reference interactomes are human PPIs. The third type of dataset was a synthetic dataset. To be precise, we used a synthetic dataset provided by the “International Network Medicine Consortium Protein–Protein Interactions Prediction Challenge”[37] that had been generated based on the gene duplication-mutation model[45].

For some primary datasets, which are not necessarily binary PPIs, we used the datasets’ annotations to extract the binary PPIs only as follows: ’physical’ for BioGRID; ’binding’ for STRING; ’direct interaction’, ’physical association’, and ’association’ for MINT. Lit-NB-13 contains non-binary PPIs only but was previously preprocessed in the study[42] so that it can be represented by binary PPIs. (This dataset of non-binary PPIs was included here in order to make a comparison to the L3 link predictor since this dataset was also used in [18].) For the reference interactomes and the synthetic dataset, all the PPIs were binary interactions. Next, every directional PPI was converted into a non-directional PPI, and all duplicate PPIs (due to multiple evidence in the literature) were excluded. The file size and the number of nodes, PPIs, candidate PPIs, and self-interacting proteins for each dataset are listed in Table 1.

### Link predictors

The L3N and L3N’ predictors, using each of the two similarity metrics $f_{1}$ and $f_{2}$ from Sect. 4.1, were compared to five other link predictors in the literature, along with an extra negative-control predictor that selected PPIs uniformly at random. Table 2 summarizes the link predictors used in the experiments. The link predictors in our study can be classified as *CN-based* or *L3-based* link predictors. The former compute $P_{xy}$ using primarily information derived from traversing the immediate common neighbor subgraph, whereas the latter iterate over $P_{4}$-subgraphs. The mechanisms of these link predictors can be summarized as follows: CN infers edges according to the principle shown in Fig. 1a; CRA infers edges using the $f_1$ similarity metric as explained in Sect. 4.1.1; L3 infers edges based on the principle shown in Fig. 1b; Sim infers edges using the $f_2$ similarity metric defined in Sect. 4.1.2; and CH2 rewards edges for which the nodes in *U* and *V* are connected to many other nodes in $U\cup V$ but not connected to many nodes outside of $U\cup V$.

**Table 2:** Overview of the link predictors used in the experiments. In the table, $A = N(x)\cap N(y)$ and $c = U\cup V$. (For the other definitions, refer to Sects. 2 and 3)

Type	Link predictor	Score function $P_{xy} =$
CN-based	Common Neighbors (CN)[20]	$|N(x)\cap N(y)|$
	CRA[32]	$\sum _{a\in A} \frac{|N(a)\cap A|}{|N(a)|}$
L3-based	L3[18]	$\sum _{U,V} \frac{1}{\sqrt{|N(u_{i})|\cdot |N(v_{j})|}}$
	CH2_L3 (CH2)[23]	$\sum _{U,V} \frac{\sqrt{(1+|N(u)\cap c|)\cdot (1+|N(v)\cap c|)}}{\sqrt{(1+|N(u)\backslash c)\backslash \{x,y\}|)\cdot (1+|N(v)\backslash c)\backslash \{x,y\}|)}}$
	Sim[33]	$\sum _{V} \frac{|N(v)\cap N(x)|}{|N(v)\cup N(x)|} + \sum _{U} \frac{|N(u)\cap N(y)|}{|N(u)\cup N(y)|}$
	NormalizedL3 (L3N) predictors	Plug in either $f_1$ or $f_2$ into Formula (6) or (7)
control	rand	Rank the edges uniformly at random

### Statistical analysis

The following standard statistical methods were employed. All statistical significance tests (p-values) were implemented by using a two-tailed independent two-sample t-test in the Python Scipy package[46]. All precision-recall (PR) curves[47] that evaluate PPI predictions were computed using the number of true-positive PPIs (*tp*), false-positive PPIs (*fp*), and false-negative PPIs (*fn*), where the precision is defined as $\frac{tp}{tp+fp}$, and the recall is defined as $\frac{tp}{tp+fn}$. The area under curve (AUC) of any arbitrary curve was computed using the trapezoidal rule in the scikit-learn package[48] in Python. In the figures presented in the following sections, error bars denote standard deviation unless otherwise stated.

## Evaluating link predictors in simulated L3 graphs

In this section, we present the results of our first set of computational validations, designed to test how well the L3N link predictors realized the L3 principle compared to the other predictors in Table 2 in some simulated scenarios. The goal here is to determine, as a starting point of our investigations, how sensitive our approach is to changes in ideal L3 graphs, as compared to the other existing link predictors. Note that since real PPI networks are not ideal L3 graphs in practice, the results in this section alone do not directly imply that our approach will work well on real datasets; the performance on real datasets will be evaluated in Sect. 7 below.

The methodology is as follows. We start with an ideal L3 graph *G* (recall the definitions from Sect. 4) consisting of two non-adjacent nodes *x* and *y*, 50 nodes in the set *U*, and 50 nodes in the set *V*. Then, in the simulations, we add or remove edges from *G* that induce changes in the $P_{xy}$-scores computed by the link predictors. By modifying an ideal L3 graph in this way, we can see the relative sensitivity of each link predictor when dealing with changes that make *G* diverge from its ideal form. From here on, an edge of the form $\{u_i, v_j\}$, where $u_i \in U$, $v_j \in V$, and $i\ne j$, will be referred to as a *compatible edge*. Similarly, an edge of the form $\{u_i, u_j\}$ where $u_i, u_j \in U$ and $u_i \ne u_j$, or of the form $\{v_i, v_j\}$ where $v_i, v_j \in V$ and $v_i \ne v_j$, or of the form $\{x, v_i\}$ where $v_i \in V$, or of the form $\{y, u_i\}$ where $u_i \in U$, is called an *incompatible edge*.

To compare different link predictors, since the range of scores for each link predictor may differ, we normalize all their scores to values between zero and one. The rand link predictor is not considered here since it cannot generate a link prediction score, so we use an alternative control predictor that simply counts the number of $P_{4}$’s between the two given nodes *x* and *y* instead. CN-based predictors are also not considered here, since their goal is not to evaluate features of $P_{4}$, so whether they are sensitive to changes in an L3 graph does not matter. (The results for the CN-based link predictors are still included in Additional file 1: Fig. S1 for completeness.)

### Removing compatible edges

Our first simulation started with the ideal L3 graph *G* and removed one of the compatible edges, chosen uniformly at random, from *G* in each iteration until all the $(50 \cdot 50)-50 = 2450$ compatible edges had been removed. Since the 50 edges of the form $\{u_{i}, v_{i}\}$ were never removed, the four L3-elements *x*, *y*, *U*, and *V* remained the same throughout the simulation. In every iteration, $P_{xy}$ for each link predictor were computed. This was repeated ten times, and the variance (in terms of the minimum and maximum values) was calculated.

**Fig. 4:**
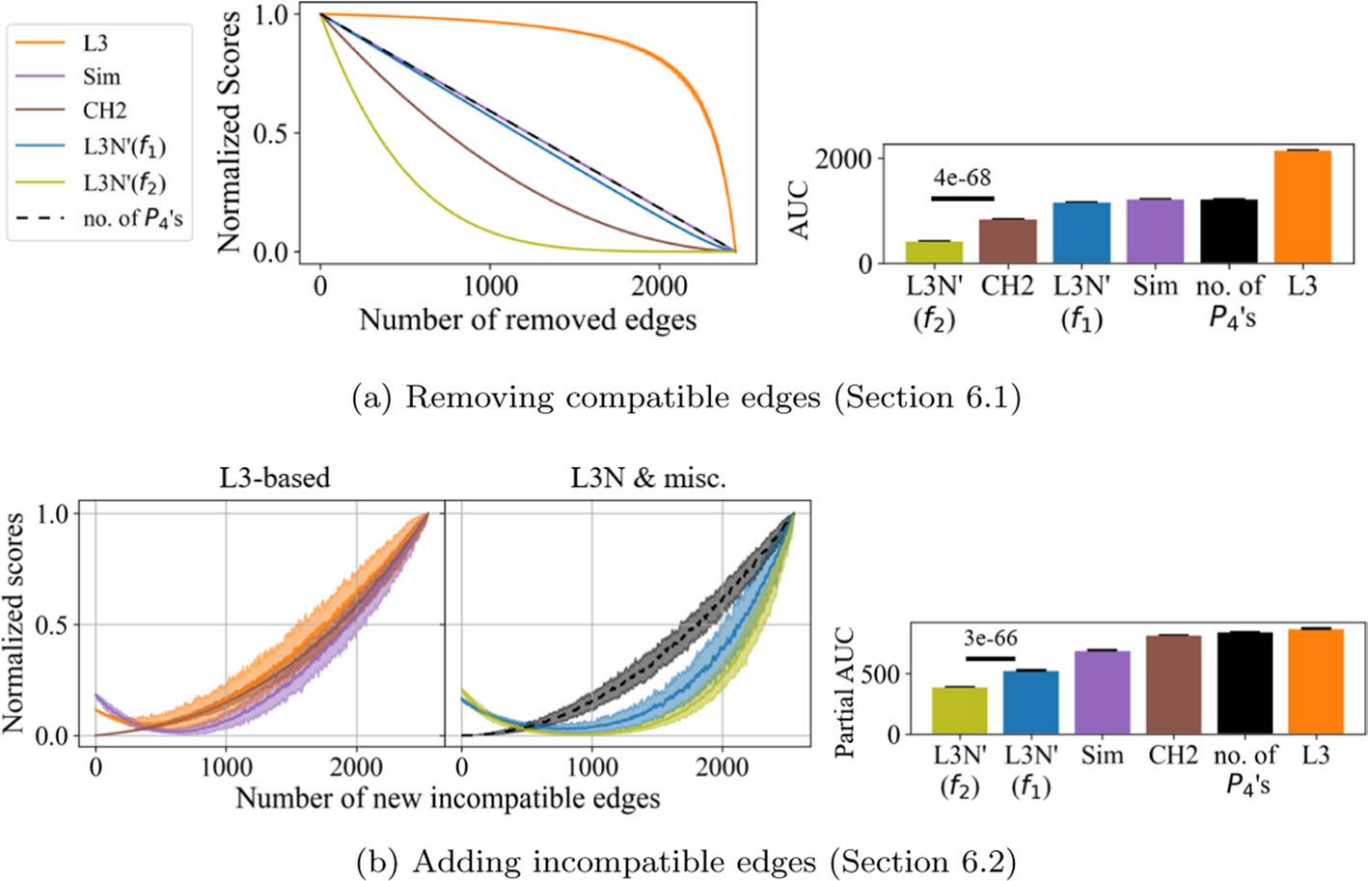
Changes in scores for different link predictors when an ideal L3 graph is modified by: **a** removing compatible edges; and **b** adding incompatible edges. The shaded regions denote the variance (the minimum and maximum values) among repeated simulations, and the solid lines denote the medians. The AUC bar charts correspond to the respective plots. In **b**, a Savitzky-Golay filter using a polynomial of degree 3 and a window size of 21 was applied to make the curves smoother

Figure 4a shows the results. (The results for L3N are plotted separately in Additional file 1: Fig. S1a since they overlap with L3N’.) As can be seen by looking at the curve for the control predictor, the number of $P_{4}$’s decreases as the number of remaining compatible edges decreases. Therefore, the $P_{xy}$-score of a well-functioning L3 link predictor should decrease at the same rate as or faster than the control predictor, achieving a smaller AUC. Here, L3N’($f_{2}$) outperformed all the other predictors, and CH2 and L3N’($f_{1}$) also did quite well. The same applies for L3N($f_1$) and L3N($f_2$) in Additional file 1: Fig. S1a.

Interestingly, L3 was less sensitive than the control predictor, and this can be attributed to the following: in early iterations, many pairs of nodes from *U* and *V* contribute to the score, and since these nodes have a high node degree, each pair has a low L3 score. Their individual contributions are consequently very small, which means that when one edge is deleted, the score computed by L3 remains close to its initial score. In contrast, in later iterations, few pairs of nodes from *U* and *V* contribute (and these nodes have a lower degree), so deleting an edge affects the score more. This indicates that the L3 link predictor fails to fully implement the L3 principle in the sense that it is insensitive to minor changes inside an ideal L3 graph with high-degree nodes.

### Adding incompatible edges

The second simulation was complementary to the one in Sect. 6.1. Starting from the ideal L3 graph *G*, one incompatible edge was inserted into *G* in every iteration until all the $$\left( {\begin{array}{c}50\\ 2\end{array}}\right) + \left( {\begin{array}{c}50\\ 2\end{array}}\right) + 50 + 50 = 2550$$ incompatible edges had been inserted. Each edge to be inserted was chosen uniformly at random among the incompatible edges that had not been inserted yet. The simulation was also repeated ten times and the results are plotted in Fig. 4b. (As above, the results for L3N are plotted separately in Additional file 1: Fig. S1b since they overlap with L3N’.)

In this simulation, one might expect to see strictly decreasing scores as additional edges are inserted into *G*, disrupting its ideal L3 structure. However, as shown by the control predictor, the addition of incompatible edges increases the number of $P_4$’s non-linearly because the more edges that already exist in *G*, the more $P_4$’s between *x* and *y* will be created for each additional edge. Therefore, any L3-based predictor will eventually show an increasing score. Yet, L3-based predictors with proper penalization should still be less sensitive than the control predictor. By this, we mean that for a link predictor, the partial AUC starting from the point on the x-axis where its minimum y-value occurs should be smaller than that of the control predictor. Here, L3N’($f_{1}$) and L3N’($f_{2}$) outperformed all the other predictors: all L3 predictors show an initially decreasing score as expected, but L3N is the least sensitive during the increase in scores as demonstrated by it having the smallest partial AUC. (The same applies for L3N($f_1$) and L3N($f_2$) in Additional file 1: Fig. S1b.) Once again, L3 had the highest AUC and was the least sensitive to changes of the form of incompatible edges being added. This is because L3 normalizes based on the node degrees rather than according to specific properties like the presence of incompatible edges. In short, the results of the simulations in this section show that among the different link predictors, L3N is the most sensitive to small changes in an ideal L3 graph.

## Link prediction in PPI datasets

In this section, we computationally evaluate the predictive power and biological significance of L3N using real PPI datasets. Applying different link predictors to datasets prepared by Monte Carlo sampling, we computed the scores of all non-neighboring pairs of nodes *x* and *y* (called *candidate edges*) and ranked them according to their scores. Unless otherwise noted, we then selected the *k* top-ranked candidate edges to be the set of predicted edges, where *k* denotes the number of edges that were removed from that dataset in the sampling preprocessing step. (In other words, the accuracy, precision, and recall would all be 100% if and only if the predicted edges were exactly those that had been removed earlier.) Finally, the performance of the various link predictors was evaluated by analyzing the sets of edges that they had predicted.

To compare the performance of link predictors, we use PR curves; see Sect. 5.3 for definitions. In general, the larger the AUC of such a curve (referred to as PR AUC from here on), the better[49].

**Fig. 5:**
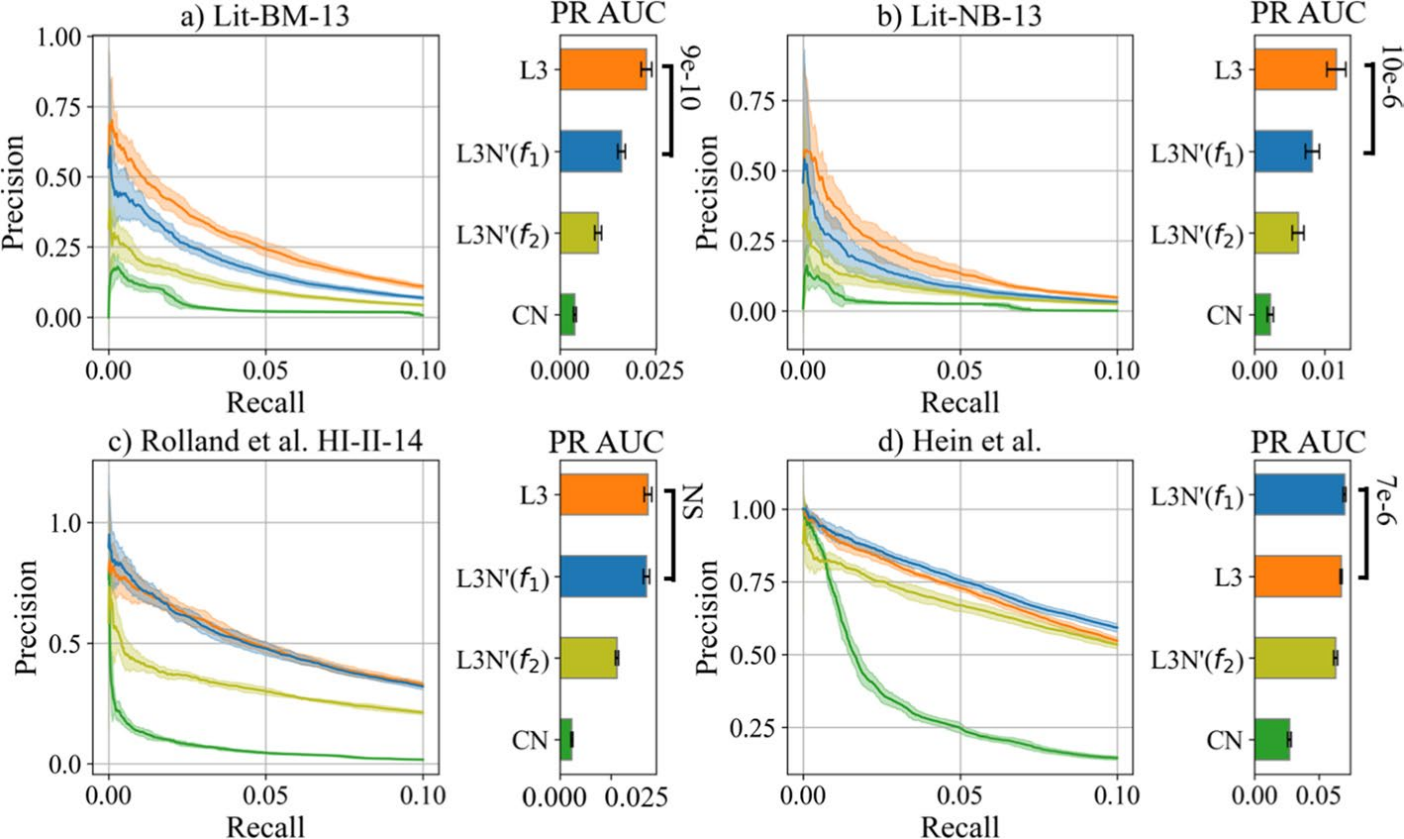
Precision-Recall (PR) curves of the link predictors computed in the datasets used in the study[18] under the same methodology (50% of the PPIs removed, computations repeated 10 times, shaded regions indicate the standard deviations, PR is calculated until the recall reaches 10%). The accompanying bar charts show the predictors’ PR AUC-values (the larger, the better)

### Comparing L3N to L3 on previous datasets

Our first computational comparison between L3 and L3N used the same datasets and conditions as the study that introduced L3[18]. To do the computational cross-validation, they first downloaded two primary datasets and two reference interactomes, and prepared ten sample datasets for each full dataset by removing 50% of the PPIs uniformly at random (Monte Carlo sampling). After obtaining the link prediction scores for all candidate PPIs, they calculated the precision and recall of the link predictors by validating each predicted PPIs in the order of their scores, until they reached a recall of 10%. Then, they compared their results with CN. In our study, we compared their results to L3N’ and the outcome is shown in Fig. 5 (results for L3N can be found in Additional file 1: Fig. S2).

According to Fig. 5, L3N’($f_{1}$) beats L3 in one case (Hein et al.), L3 beats L3N’($f_{1}$) in two cases (Lit-BM-13 and Lit-NB-13), and L3N’($f_{1}$) and L3 exhibit a similar performance in one case (HI-II-14). Considering that L3N evaluates *xy* node pairs using not only the properties of existing PPIs, but also PPIs that are absent, PPI networks that are highly incomplete will likely give false information to L3N, leading to the predictions being ineffective. This would explain why L3N performs slightly better in Fig. 5d), corresponding to the dataset with the most PPI coverage among the four. The next subsection will compare the link predictors’ performance on various datasets that systemically curated PPIs from the literature.

**Fig. 6:**
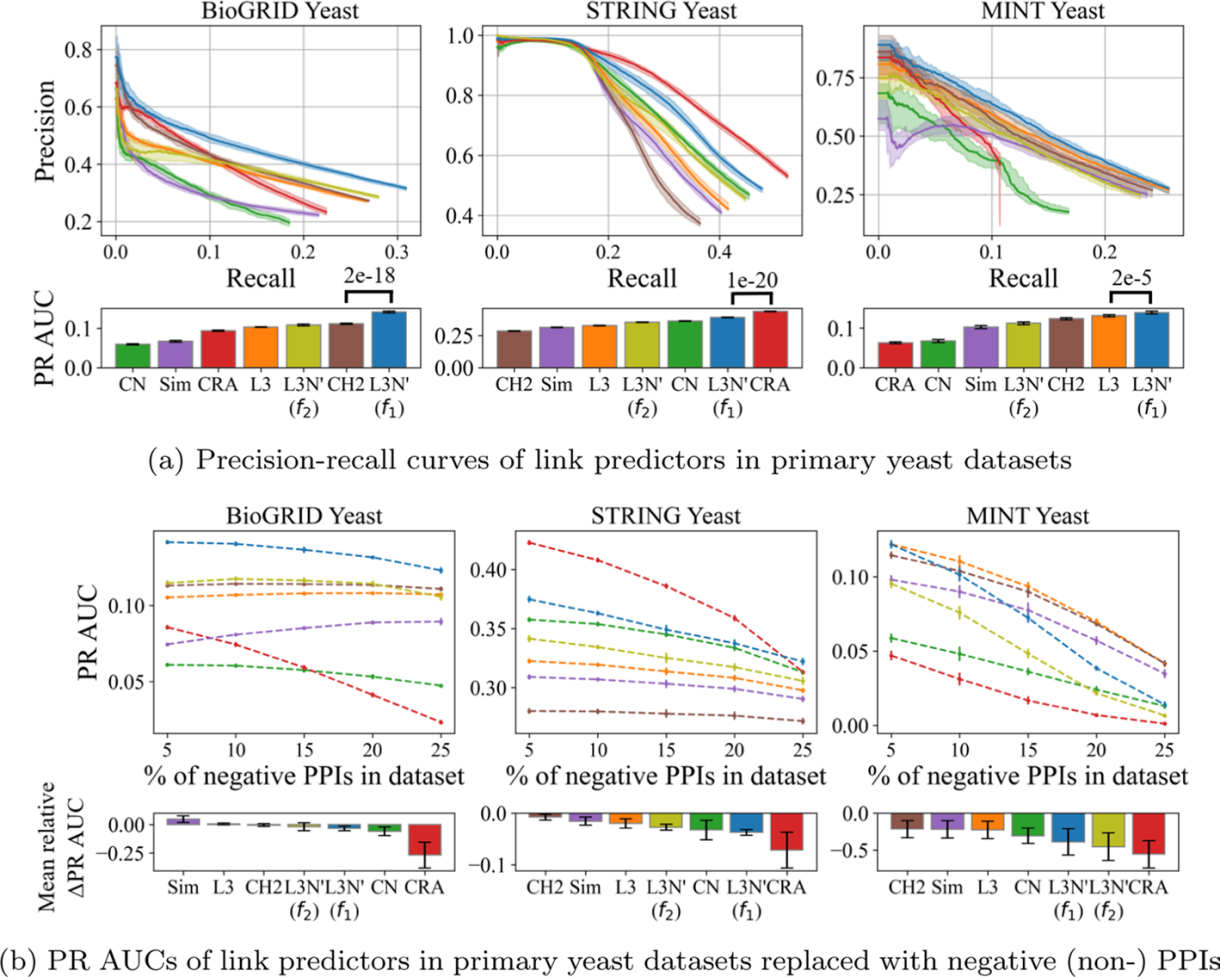
Precision-recall (PR) curves and its AUC-values (PR AUCs) of the link predictors computed with **a** 50% of the PPIs removed in the datasets. The solid lines show the median values and the shaded regions indicate the variance (the minimum and maximum values). The accompanying bar charts show the predictors’ PR AUC-values (the larger, the better). **b** Using these datasets, either 5%, 10%, 15%, 20%, or 25% of the PPIs are replaced with negative (non-) PPIs. For each these datasets, the PR AUCs computed by a link predictor are extracted at each data point and interpolated as a dotted line. The mean relative change of PR AUC w.r.t. the changes in the ratio of negative PPIs are obtained and denoted as “mean relative $\Delta$PR AUC” (the lower, the worse)

### Statistical significance of L3N link predictions in primary datasets

Motivated by the results above, we conducted further computational experiments under the same setup using three primary datasets of yeast: BioGRID, STRING, and MINT. To expand the scope, the other link predictors mentioned in Sect. 5.2 were also included. Figure 6a shows the PR curves and PR AUC-values of the link predictors. Note that the results for the negative-control link predictor (rand) have been deferred to the Additional file 1 and omitted from Fig. 6 because its PR AUCs are too insignificant to visualize. We removed self-interacting proteins (SIPs) in these datasets because we are only interested in binary PPIs of different proteins. For completeness, the SIPs have been included in Additional file 1: Fig. S3 and it can be seen that the difference to Fig. 6a is minimal because the number of SIPs is actually very small (see Table 1). Comparing the results in Fig. 6a to Fig. 5, we found that now L3N’ not only performs better than L3 in all scenarios, but also better than most of the other link predictors in terms of PR AUC. Moreover, L3N’ is slightly better than L3N (see Additional file 1: Fig. S4), so we will focus on L3N’ from here on.

#### Adding false information into primary datasets

In Sect. 7.1, we hypothesized that false information may affect the performance of L3N, so we further investigated this issue by modifying the sampled datasets above by replacing 5%, 10%, 15%, 20%, and 25% of the positive (real) PPIs with negative (non-) PPIs and then performing link predictions in the same way. Figure 6b shows the PR-AUC of each such dataset plotted against the percentage of PPIs replaced by negative PPIs. (Full PR curves in Additional file 1: Fig. S5, the p-values of all the predictors against the rand predictor in Additional file 1: Table S2, and the PR AUC of the rand predictor in Additional file 1: Table S3). As shown in the figure, the PR AUC of most link predictors decrease as there are more negative PPIs in the datasets, which is an expected outcome since the scores of the candidate PPIs are computed based on more and more false information. The relative performance of different link predictors is also similar to that of Fig. 6a, and the precision appears to remain largely unaffected compared to the recall (Additional file 1: Fig. S5). However, examining the mean changes of PR AUC w.r.t. the changes in the ratio of negative PPIs (abbreviated as $\Delta$AUC) reveals some interesting patterns. In short, a negative $\Delta$AUC means the PR AUC generally decreases as there are more negative PPIs in the datasets. Therefore, a low $\Delta$AUC implies the link predictor being more sensitive to negative PPIs and the quality of its prediction drops. Undoubtedly, among the L3 predictors, L3N has the highest $\Delta$AUC across all three datasets, suggesting that indeed false information influence L3N more due to our approach, where we exhaustively evaluate conditions that constitute an ideal L3 graph.

**Fig. 7:**
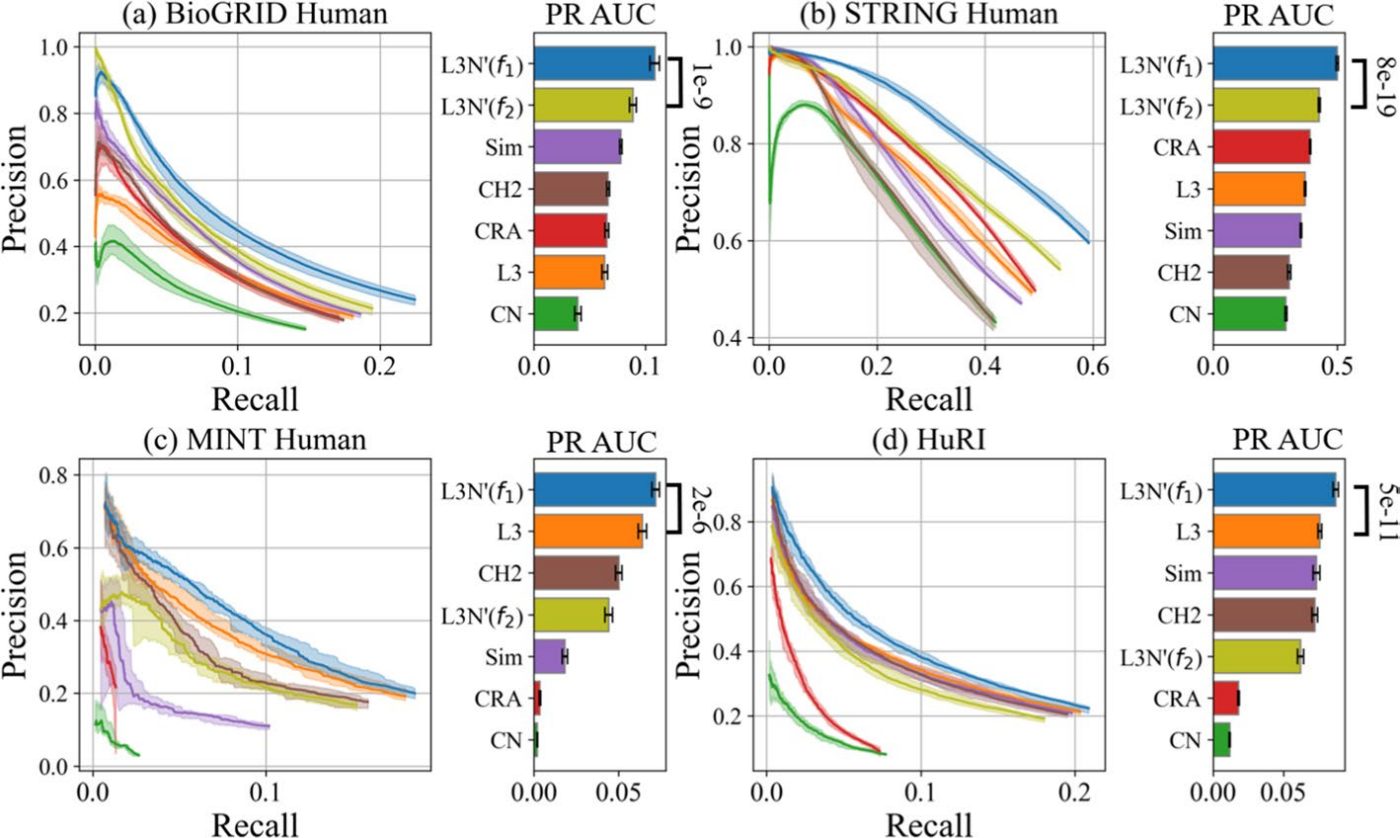
Precision-Recall (PR) curves of the link predictors using the same experiment setup as in Fig. 6a, except here the datasets are human datasets: **a**–**c** primary human datasets and **d** a human reference interactome. Specifically, the setup is to remove 50% of PPIs for each datasets for ten times randomly at uniform to generate ten sample datasets, where the shaded regions illustrate the variance in terms of minimum and maximum value, solid line as the median, and the accompanying bar charts for PR AUC-values (the larger, the better)

#### L3N link predictions in other human datasets

Next, we further expanded our analysis using the same setup as in Sect. 7.1 and using the remaining primary human datasets (BioGRID, STRING, and MINT) and a human reference interactome (HuRI), which is an updated version of the four datasets used in Fig. 5. As shown in Fig. 7, L3N’ has the best overall performance among the link predictors on the yeast datasets. This further supports our claim that L3N is good at predicting PPIs.

We also tested a synthetic dataset from a recent International PPI challenge[37]. Due to the way that the dataset was modeled and generated, L3-based predictors have a significant advantage over CN-based predictors and L3N was indeed the top predictor (see Additional file 1: Fig. S6).

**Fig. 8:**
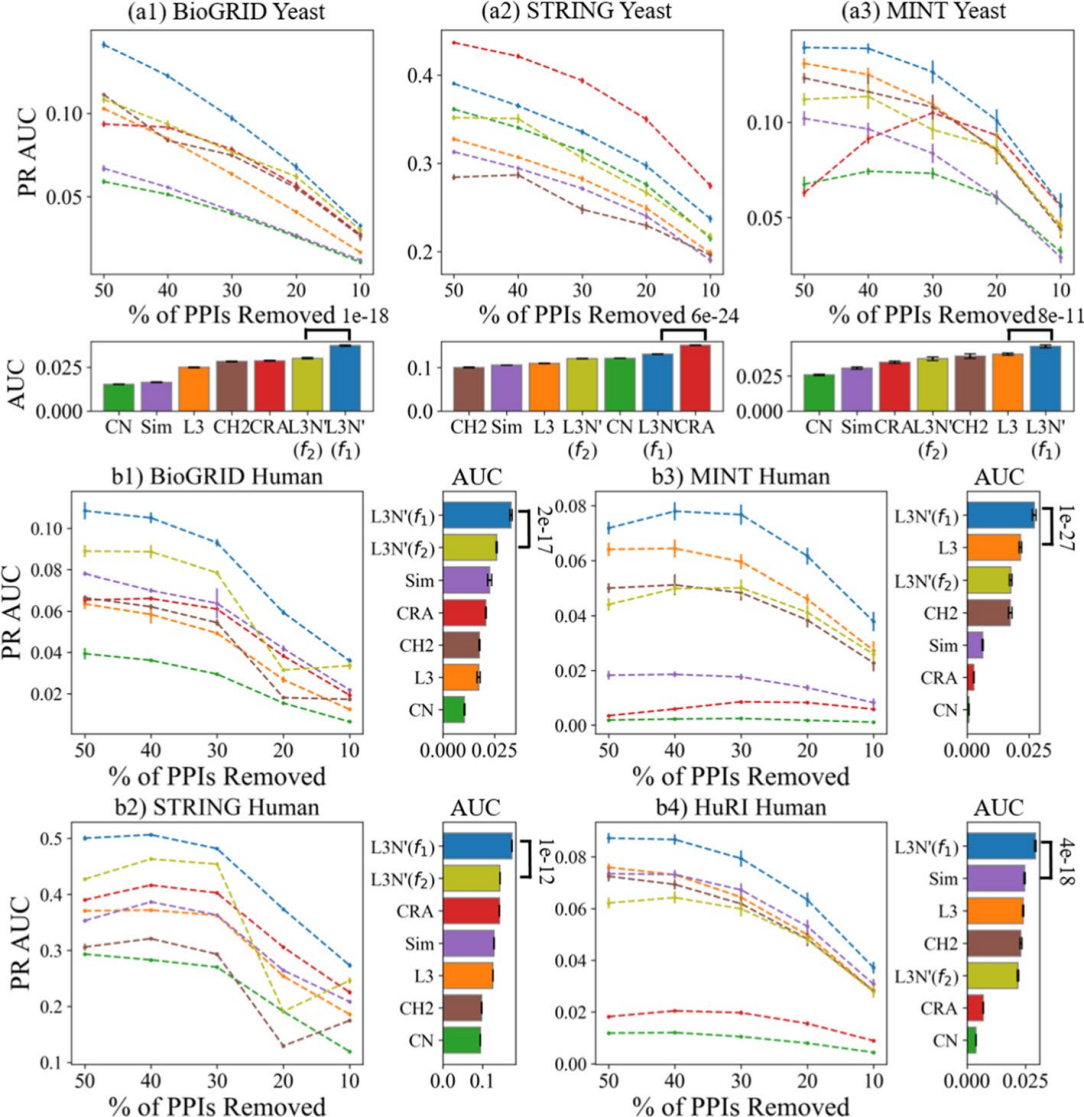
Illustrating how the PR AUC of the link predictors changes as the percentage of PPIs removed from the datasets decreases. The dotted curves are interpolations of the data points (50%, 40%, 30%, 20%, and 10%). The bar charts show the AUCs of the PR AUCs, i.e., the total area under each dotted line (the larger, the better)

#### L3N link predictions in different sizes of datasets

In Sect. 7.1, we mentioned that PPI coverage of a dataset could affect the performance of PPI link prediction. While we cannot assess the influence of PPI coverage on link predictions since we cannot know in advance how many of the PPIs remain undiscovered, we can nevertheless validate how different sizes of PPI networks affect link prediction. We ran the computational validations again on all the datasets using the same setup in Fig. 6a, except this time instead of removing 50% of the edges for Monte Carlo sampling, we removed either 40%, 30%, 20%, or 10% of the edges to prepare the sample datasets. The computed PR curves and PR AUC-values are plotted in Additional file 1: Figs. S7–S10, and the outcome is similar to that of Fig. 6a. (The p-values of the PR AUC of all predictors against the rand predictor in Additional file 1: Table S1 and S4.) To give a summary of Additional file 1: Figs. S7–S10, we extracted the PR AUC for each of the predictors in the computational experiments, and plotted them in Fig. 8 to show the changes in PR AUC as the number of edges removed in the dataset decreases. As shown in Fig. 7, L3N’($f_1$) outperforms all the other link predictors in most datasets with high statistical significance, indicating that L3N remains a good predictor as the size of the datasets grow. Another observation is that the PR AUC along the x-axis decreases, which may be because of the rapid drop in precision-recall or the drop in maximum recall as the percentage of removed edges in the datasets decreases (see Additional file 1: Figs. S7–S10). To investigate the reason for this, we evaluated the PR AUC of the random predictor as a negative control (Additional file 1: Table S5). There is a gradual decrease in the PR AUC as the number of removed PPIs decreases, suggesting that if fewer PPIs are removed then it is more difficult for a predictor to pick a real PPI at random.

**Table 3:** Average computation times (in minutes) with standard deviation (denoted by ±) for the experiments in Fig. 7

CN	CRA	L3	CH2_L3	Sim	L3N’($f_{1}$)	L3N’($f_{2}$)
*BioGRID yeast*						
$2.65\pm 0.06$	$2.51\pm 0.03$	$5.76\pm 0.08$	$46.9\pm 1.54$	$12.4\pm 0.24$	$11.9\pm 0.55$	$52.8\pm 1.18$
*STRING yeast*						
$1.22\pm 0.11$	$1.2\pm 0.01$	$2.96\pm 0.88$	$10.3\pm 0.71$	$3.81\pm 0.07$	$6.35\pm 0.46$	$13.0\pm 0.6$
*MINT yeast*						
$0.82\pm 0.03$	$0.81\pm 0.01$	$0.88\pm 0.01$	$0.91\pm 0.03$	$0.91\pm 0.02$	$0.9\pm 0.02$	$0.98\pm 0.06$
*BioGRID human*						
$1.25\pm 0.01$	$1.12\pm 0.04$	$16.1\pm 0.37$	$117\pm 4.78$	$35.5\pm 3.6$	$27.9\pm 0.89$	$130\pm 5.00$
*STRING human*						
$0.42\pm 0.01$	$0.45\pm 0.01$	$6.72\pm 0.09$	$25.3\pm 0.5$	$9.61\pm 0.17$	$12.0\pm 0.19$	$31.5\pm 0.71$
*MINT human*						
$2.03\pm 0.14$	$1.93\pm 0.02$	$2.12\pm 0.11$	$2.17\pm 0.02$	$2.12\pm 0.02$	$2.13\pm 0.02$	$2.27\pm 0.04$
*HuRI human*						
$2.87\pm 0.1$	$2.73\pm 0.03$	$3.28\pm 0.09$	$3.6\pm 0.06$	$3.41\pm 0.04$	$3.41\pm 0.04$	$3.74\pm 0.04$
*Synthetic*						
$3.57\pm 0.07$	$3.38\pm 0.01$	$3.85\pm 0.03$	$4.13\pm 0.33$	$4.92\pm 0.07$	$4.92\pm 0.05$	$4.3\pm 0.14$

#### Computation time of link predictors

In addition to the predictive power, another important aspect to consider in the evaluation of link predictors is the computation time. Table 3 summarizes the computation times taken by the experiments in Fig. 7. The experiments were conducted using a setup of 14 cores and 32GB RAM. A larger setup consisting of 24 cores and 128GB RAM was used for the BioGRID Human and STRING Human datasets due to their massive size. For L3N’, the computation time increases more rapidly than for the simpler predictors CN, L3, and CRA as the datasets scale up (e.g., BioGRID Yeast and Human), in accordance with the time complexity analysis in Sect. 4.3. Yet, if L3N’ is compared to other non-trivial L3-based predictors (CH2, Sim), it can be seen that L3N’($f_1$) is able to obtain larger AUC-values (sometimes twice as large, according to Fig. 7) using much less time than CH2 and roughly the same amount of time as Sim. As for L3N’($f_2$), it’s slower in practice than L3N’($f_1$) although both methods have the same theoretical time complexity (see Sect. 4.3).

#### Influence of data sampling to PPI link predictions

The final aspect that we considered here was the sampling approach used to prepare our datasets. We employed a Monte Carlo sampling approach, varying the sampling size (50%, 60%, 70%, 80%, and 90%), but another popular sampling approach is the K-Fold cross-validation (CV)[16]. To test whether the sampling approach has a significant effect on the performance, we tested a subset of the datasets (BioGRID Yeast, STRING Yeast, MINT Yeast) using the 5-fold CV. As shown in Additional file 1: Fig. S11, since 5-fold CV samples 80% of the dataset as the training set, it leads to a similar performance as in Additional file 1: Fig. S9, which also samples 80% of the datasets (except that it is repeated ten times). This demonstrates that the choice of sampling approach has a minimum impact on the final outcome.

Overall, the above findings lead us to conclude that L3N’($f_1$) has the best predictive power in terms of precision-recall across datasets of different sample sizes, with trade-offs in terms of the influence from false information (negative PPIs), at the cost of an increase in the computation time in some of the cases.

### PPI confidence scores for L3N predictions in primary datasets

**Fig. 9:**
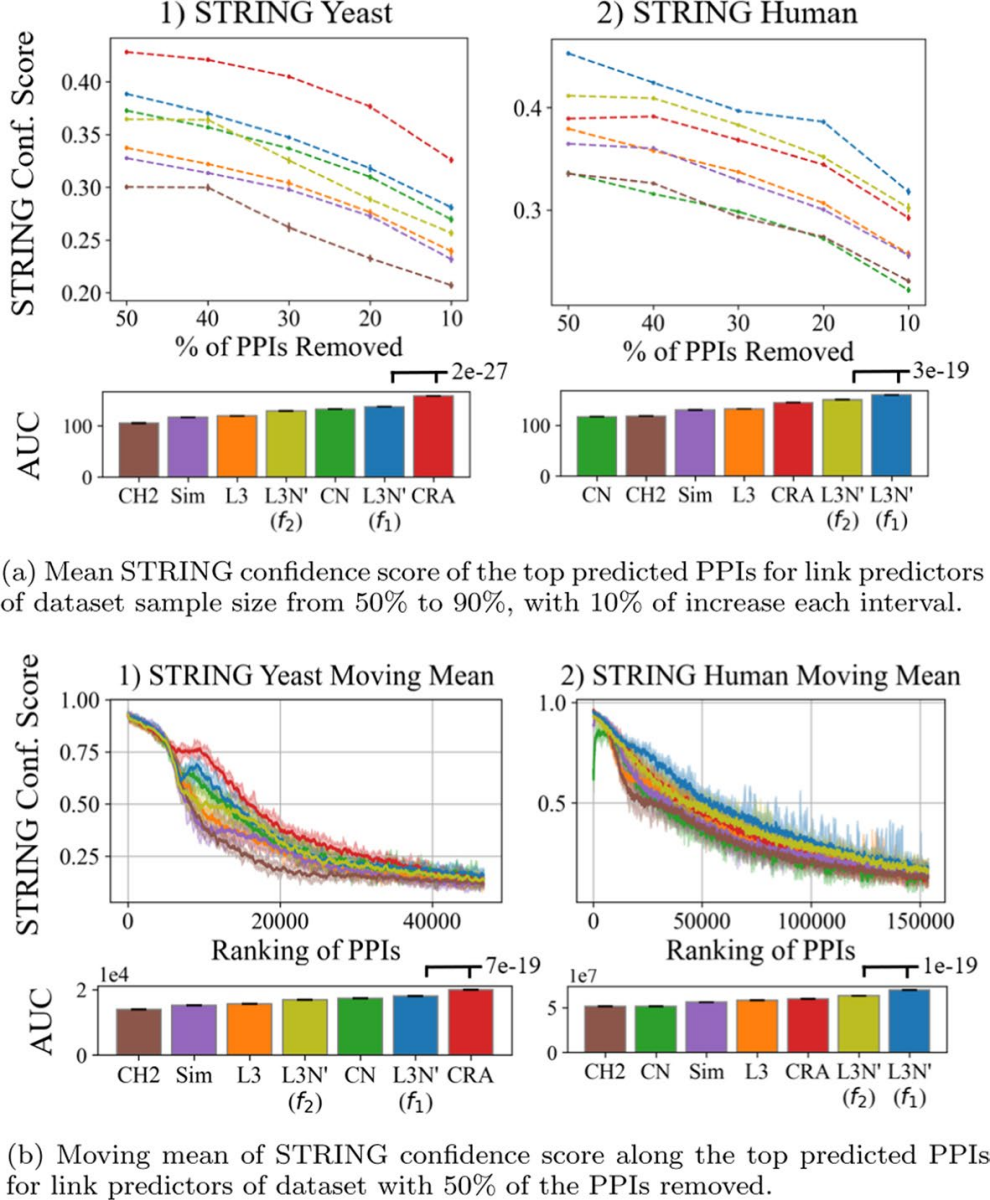
**a** The mean STRING confidence score across all sample sizes, and **b** the moving means of the STRING confidence scores for sample size 50%. The shaded regions in Fig. 8b illustrate the variance (the minimum and maximum values) in STRING confidence scores

In addition to the statistical significance of L3N’ in PPI link prediction, we are also interested in computationally validating the biological evidence of the predicted PPIs. The first measure that we consider is the STRING confidence score[39]. The STRING confidence score estimates the confidence of a PPI by evaluating evidence for the two proteins such as whether their genes co-express, whether the proteins co-occur phylogenetically, whether the proteins appear together frequently in the literature, and more. We extracted the STRING confidence scores from the STRING datasets, interpreting every null score as a zero. Figure 9a shows the mean STRING confidence scores of the predicted PPIs across different sample sizes of all datasets for every predictor. The random predictor (rand) has been omitted from the figure due to its insignificance. According to the plots, L3N’ is the best predictor for the human dataset and the second-best predictor for the yeast dataset (after CRA). This validates computationally that PPIs predicted by L3N’ are biologically relevant.

To investigate whether there is a correlation between the ranking of a predicted PPI and its STRING confidence score, we plotted the moving mean (window size of 100, 10 steps forward in each iteration) of the STRING confidence score along with the ranking of the predicted PPIs in Fig. 9b for datasets with 50% of the PPIs removed. The figures for the rest of the sample sizes are included in Additional file 1: Figs. S12 and S13. The moving mean shows that for every predictor, the predicted PPIs that are ranked higher indeed have a higher STRING confidence score than those that are ranked lower. The difference between L3N’ and the other predictors is that, like in the situation in Fig. 9a, L3N’ is the best predictor for the human dataset where the predicted PPIs in general have higher confidence scores, and the second-best predictor for the yeast dataset.

Next, we computed the gene ontology (GO) Semantic Similarity (GOSemSim) scores of the predicted PPIs. The GOSemSim score estimates the similarity of two proteins based on the similarity of their so-called GO annotations that describe proteins in terms of their role as a cellular component, their role in molecular functions, and their role within biological processes. The implementation that we used was a GOSemSim package written in the R programming language [50] based on Wang’s method [51] with the BMA strategy; null GOSemSim scores are ignored in the computations. Additional file 1: Fig. S14 shows the GOSemSim scores of the predicted PPIs of all predictors across the datasets of different sample sizes. The link predictors are separated for comparison according to the principle they are based on (Table 2: CN-based, L3-based, or control). While the differences between predictors are less striking than in the experiments above, we can observe that the CN-based predictors have better GOSemSim scores than the L3-based predictors in general. This is natural because CN characterizes protein pairs with similar functions (Sect. 2.1). Among the L3-based predictors, we can see that L3N’ beats the others with statistical significance (in terms of AUC-values using student’s t-test) for four of the seven datasets. Hence, PPIs that are ranked highly by L3N’ may possess some functional bias that is encouraged by GOSemSim, e.g., physical PPIs with high L3 scores may reside in neighboring cellular components.

### Differences in PPIs predicted by CRA and L3N

The CRA predictor, a CN-based predictor, is one of the best link predictors in the above computational validations but with a huge variance in its performance. For example, it appears to outperform L3N in some datasets as shown in Fig. 8 and Fig. 9, but it is also easily influenced by negative PPIs as shown in Fig. 6b. We hypothesized that this is due to the different paradigms adopted by L3N and CRA in their respective network modeling, so we further investigated the similarity between the pools of PPIs predicted by CRA and L3N’($f_{1}$), i.e., the ratio of the overlap. Surprisingly, as shown in Table 4, these two predictors show a lower overlap ratio compared to the mean overlap ratios of L3-based predictor pairs or CN-based predictor pairs. A lower overlap ratio can also be seen even if we compute overlap ratios of pairs where one predictor is CN-based and another is L3-based predictor. This implies that the PPIs predicted by L3N are similar to those predicted by other L3 predictors, although L3N is better at ranking them (see Sect. 7.2). Furthermore, this suggests that since L3N and CRA predict differing sets of PPIs with competing performance based on different assumptions, the two methods could perhaps be used together in a complementary way to obtain even better link predictions.

**Table 4:** Overlap ratios of predicted PPIs between different types of link predictors for datasets with 50% of the PPIs removed (Table S6 and S7 show the complete data)

Overlap ratio of predicted PPIs between types of link predictors				
Dataset \overlap between	CN-based	L3-based	CN & L3-based	CRA & L3N’($f_{1}$)
BioGRID Yeast	69%	79 ± 10 %	30 ± 6 %	35%
STRING Yeast	89%	92 ± 2 %	72 ± 6 %	74%
MINT Yeast	43%	72 ± 8 %	32 ± 2 %	34%
BioGRID Human	64%	69 ± 4 %	38 ± 4 %	37%
STRING Human	54%	58 ± 4 %	44 ± 3 %	44%
MINT Human	37%	71 ± 10 %	4 ± 2 %	5%
HuRI	64%	79 ± 7 %	24 ± 3 %	23%

’CN-based’ and ’CRA & L3N’($f_{1}$)’ denotes the overlap ratio of the predicted PPIs between CN and CRA, and between CRA and L3N’($f_{1}$) respectively. For ’L3-based’, since there are multiple L3-based predictors (L3, CH2, Sim, L3N’($f_{1}$), and L3N’($f_{2}$)), they calculated the overlap ratio for each pair of predictors. We then took the mean of these ratios as the final value, and also computed the standard deviation. The same applies to ’CN & L3-based’ where a CN predictor is compared to an L3-based predictor. Blue color denotes a relatively higher overlap ratio and red a relatively smaller overlap. Ratios are rounded to nearest integers

## Discussion

We have proposed a way to implement the L3 principle in link predictors that we call NormalizedL3 (L3N). Using the L3N predictors, we are able to deal with hypothetical PPI subgraphs much better than other L3 link predictors (Sect. 6). L3N can also predict PPIs with strong statistical significance (Sect. 7.2) and sufficient biological relevance (Sect. 7.3). In summary, we have demonstrated that the L3N predictors are effective predictors of missing protein–protein interactions that are better than previous methods.

The modeling strength of L3N comes from two main ideas, the realization that the L3 principle can be decomposed into a series of computations that compare graph neighborhoods, and that these comparisons can be computed using similarity metrics. These address what the other L3 predictors are lacking: the original L3 predictor[18] simplifies the L3 principle without sufficiently considering the compatibility of protein interfaces; the CH2 predictors[23] merely adopt the modeling approach of the CRA predictor in L3 subgraphs, which again does not address protein compatibility; and the Sim predictor[33] models protein compatibility using the Jaccard coefficient but only partially since it lets the sets *U* and *V* contribute to the final score independently, ignoring the biological motivation of the L3 principle (see also Fig. 1d).

Apart from the improved link prediction performance of L3N, these predictors can also be used as a heuristic to narrow down candidate proteins for biological problems. A study by Liu et al.[22] improves protein folding recognition by constructing a protein similarity network based on the L3 principle to identify proteins that could fold in similar ways as the query protein. Since adding network data yields better performance than using protein sequence and profile data only, we believe that L3N could also be used in other similar scenarios.

The focus of the present study was CN- and L3-based link predictors. In general, PPI link predictors are not limited to these principles, nor do they have to rely on topological properties only; e.g., as mentioned in Sect. 2, there are methods besides iterating and scoring candidate edges. However in PPI link predictions, prior biological knowledge is perhaps more important to obtain accurate characterizations. For example, if the goal is to reveal undiscovered PPIs based on high-throughput binary PPI data, it is important to also ensure the reliability of the underlying data. To this end, advances have been made integrating node-centric data like protein sequence data[52] and gene ontology data[53] to improve PPI prediction. The idea here is that, instead of experimentally evaluating each candidate PPI to complete the PPI interactome, we focus on generating data related to the proteins (nodes) and use this data to help predict PPIs computationally, which could be less time-consuming as there are more candidate edges to check than nodes to check. For example, protein sequence data can also be generated by high-throughput methods, which can provide supporting data for PPI predictions in a timely manner.

We anticipate that the use of biological network data will become even more prevalent in various biological problems. Therefore, methods such as L3N may turn out to be useful for many other applications beyond link prediction in protein–protein interaction networks in the future.

## Supplementary Information

**Additional file 1**. The additional file contains supplementary information of: Table S1-p-values of the computation in Fig. 6(a); Table S2-p-values of the computation in Fig. 6(b); Table S3-PR AUCs of the rand link predictor for the computation in Fig. 6(b); Table S4-p-values of the computation in Fig. 7; Table S5-PR AUCs of the rand link predictor in all the PPI datasets varying ratios of PPIs removed; Table S6 and S7-supplementary data for Table 4 of other sampling sizes of the PPI datasets; Fig. S1-supplementary data including other link predictors that is not illustrated in Fig. 4; supplementary data including other L3N link predictors results that were not illustrated in Fig. 5; Fig. S3-supplementary data of Fig. 6 with SIPs included in the datasets; Fig. S4-supplementary data including other L3N link predictors results that were not included in Fig. 6(a) and 7; Fig. S5-full PR curves of link predictors computing the datasets in Fig. 6(b); Fig. S6-PR curves and its AUC of link predictors computing the synthetic dataset; Fig. S7, S8, S9, S10-Full PR curves of link predictors computing the datasets in Fig. 8; Fig. S11-PR curves and its AUC for link predictors computing datasets sampled with 5-Fold CV; Fig. S12, S13-full moving mean STRING curves and its AUCs for link predictors computing the datasets in Fig. 9(a); Fig. S14-GOSemSim scores of link predictors computed as the percentage of removed PPIs decreases.

## Data Availability

The algorithms and the scripts written to generate and extract the data for experiments, and a command-line program to use *NormalizedL3* are all included in the following GitHub repository: https://github.com/andy897221/BMC_PPI_L3N.

## Abbreviations

PPIs: Protein–protein interactions
CN: Common neighbor
L3N: NormalizedL3
L3: Path of length-3
PR: Precision-recall
AUC: Area under curve
PR AUC: Area under precision-recall curve
conf. score: Confidence score
GO: Gene ontology
GOSemSim: Gene ontology semantic similarity
