# Supplementary material for: Normalized L3-based link prediction in protein–protein interaction networks
Source: BMC Bioinformatics. 2023 Feb 22;24:59. doi: 10.1186/s12859-023-05178-3 (PMC9945744; doi:10.1186/s12859-023-05178-3)
Supplement: Supplementary file 1 — Additional file 1. The additional file contains supplementary information of: Table S1-p-values of the computation in Fig. 6(a); Table S2-p-values of the computation in Fig. 6(b); Table S3-PR AUCs of the rand link predictor for the computation in Fig. 6(b); Table S4-p-values of the computation in Fig. 7; Table S5-PR AUCs of the rand link predictor in all the PPI datasets varying ratios of PPIs removed; Table S6 and S7-supplementary data for Table 4 of other sampling sizes of the PPI datasets; Fig. S1-supplementary data including other link predictors that is not illustrated in Fig. 4; supplementary data including other L3N link predictors results that were not illustrated in Fig. 5; Fig. S3-supplementary data of Fig. 6 with SIPs included in the datasets; Fig. S4-supplementary data including other L3N link predictors results that were not included in Fig. 6(a) and 7; Fig. S5-full PR curves of link predictors computing the datasets in Fig. 6(b); Fig. S6-PR curves and its AUC of link predictors computing the synthetic dataset; Fig. S7, S8, S9, S10-Full PR curves of link predictors computing the datasets in Fig. 8; Fig. S11-PR curves and its AUC for link predictors computing datasets sampled with 5-Fold CV; Fig. S12, S13-full moving mean STRING curves and its AUCs for link predictors computing the datasets in Fig. 9(a); Fig. S14-GOSemSim scores of link predictors computed as the percentage of removed PPIs decreases. [file 12859_2023_5178_MOESM1_ESM.pdf]

Supplementary Information: Normalized L3-Based Link Prediction in Protein-Protein Interaction Networks

Ho Yin Yuen, Jesper Jansson

	CN	CRA	L3	CH2_L3	Sim	**L3N'(f_1)**	**L3N'(f_2)**
**BioGRID Yeast**							
50% PPIs removed	4e-27	1e-29	1e-34	3e-34	4e-26	1e-31	5e-29
40% PPIs removed	1e-28	2e-29	1e-34	4e-31	4e-30	6e-33	2e-27
30% PPIs removed	5e-27	7e-29	6e-29	10e-33	10e-28	8e-30	3e-26
20% PPIs removed	8e-26	3e-24	1e-25	8e-24	2e-26	2e-25	10e-26
10% PPIs removed	1e-20	2e-22	1e-21	4e-16	3e-23	1e-23	1e-22
**STRING Yeast**							
50% PPIs removed	9e-38	5e-42	5e-37	1e-33	3e-36	3e-39	2e-36
40% PPIs removed	6e-38	1e-37	2e-37	6e-32	2e-36	2e-36	2e-31
30% PPIs removed	1e-35	6e-36	2e-33	9e-29	4e-35	4e-35	6e-31
20% PPIs removed	8e-33	7e-34	7e-31	3e-29	9e-31	5e-31	1e-30
10% PPIs removed	10e-31	6e-32	1e-29	1e-29	6e-30	2e-30	3e-30
**MINT Yeast**							
50% PPIs removed	1e-28	4e-25	1e-28	2e-28	2e-24	8e-28	3e-26
40% PPIs removed	1e-27	2e-26	1e-26	7e-19	5e-25	6e-30	4e-21
30% PPIs removed	7e-24	3e-24	8e-25	1e-20	1e-20	1e-22	3e-21
20% PPIs removed	1e-20	6e-20	3e-22	9e-18	3e-21	6e-21	2e-17
10% PPIs removed	3e-18	6e-20	2e-16	1e-16	2e-16	5e-16	2e-17

Table S1: The link predictors' p-values against negative control (the random link predictor, rand) in terms of PR AUC for varying sample sizes of the yeast datasets (ten trials). All link predictors show to have statistical significance against the PR AUC of rand, confirming that any link predictor is far better than selecting PPIs at random.

	CN	CRA	L3	CH2_L3	Sim	**L3N'(f_1)**	**L3N'(f_2)**
**BioGRID Yeast**							
5% of PPI replaced	2e-29	2e-30	5e-37	5e-34	1e-31	4e-35	8e-31
10% of PPI replaced	5e-29	2e-28	2e-34	1e-34	7e-32	2e-33	8e-33
15% of PPI replaced	2e-26	2e-26	2e-34	7e-34	2e-38	2e-31	3e-31
20% of PPI replaced	5e-26	1e-23	1e-36	7e-35	3e-31	2e-35	5e-30
25% of PPI replaced	8e-28	8e-21	4e-34	1e-31	7e-27	1e-30	8e-28
**STRING Yeast**							
5% of PPI replaced	2e-38	5e-40	6e-38	1e-35	1e-37	5e-37	8e-36
10% of PPI replaced	9e-39	1e-39	3e-40	1e-36	2e-40	2e-39	3e-39
15% of PPI replaced	3e-37	3e-38	3e-34	8e-33	4e-33	5e-34	3e-32
20% of PPI replaced	4e-36	4e-37	1e-35	4e-33	9e-35	5e-35	3e-34
25% of PPI replaced	6e-38	1e-39	6e-37	9e-35	2e-35	6e-35	2e-33
**MINT Yeast**							
5% of PPI replaced	5e-22	1e-19	2e-29	1e-28	5e-26	3e-28	4e-25
10% of PPI replaced	1e-16	3e-14	3e-24	3e-25	4e-22	3e-23	1e-20
15% of PPI replaced	1e-19	4e-13	2e-25	4e-23	6e-20	8e-23	1e-19
20% of PPI replaced	5e-17	5e-14	2e-25	5e-27	6e-22	7e-23	8e-19
25% of PPI replaced	3e-14	4e-07	3e-22	9e-22	5e-18	2e-13	1e-11

Table S2: The link predictors' p-values against negative control (the random link predictor, rand) in terms of PR AUC in the yeast datasets (ten trials). The datasets are prepared by first removing 50% of the PPIs as the sample datasets, then some ratios of the PPIs are replaced with negative PPIs. All link predictors show to have statistical significance against the PR AUC of rand, confirming that any link predictor is far better than selecting PPIs at random.

**AUC of PR for rand predictor**					
dataset \% of PPIs replaced	5%	10%	15%	20%	25%
BioGRID Yeast	7.018e-06	7.537e-06	7.641e-06	9.318e-06	8.749e-06
STRING Yeast	2.295e-05	2.544e-05	2.563e-05	2.720e-05	3.049e-05
MINT Yeast	1.750e-06	1.61e-06	1.996e-06	2.221e-06	1.789e-06

Table S3: The rand link predictor's mean PR AUC-values after some ratios (either 5%, 10%, 15%, 20%, 25%) of PPIs getting replaced with negative PPIs from the datasets (ten trials for each).

	CN	CRA	L3	CH2_L3	Sim	**L3N'(f_1)**	**L3N'(f_2)**
**BioGRID Human**							
50% PPIs removed	2e-19	1e-26	6e-24	6e-30	2e-32	4e-24	3e-25
40% PPIs removed	8e-28	4e-31	7e-19	2e-28	4e-31	1e-27	2e-24
30% PPIs removed	2e-27	9e-32	3e-29	7e-26	8e-16	1e-29	7e-32
20% PPIs removed	3e-28	5e-27	4e-21	5e-23	1e-24	2e-28	9e-26
10% PPIs removed	3e-20	1e-17	8e-21	2e-21	3e-27	2e-25	7e-21
**STRING Human**							
50% PPIs removed	3e-34	5e-39	1e-35	4e-29	5e-35	1e-35	2e-36
40% PPIs removed	5e-37	2e-40	9e-39	3e-37	1e-36	2e-38	4e-38
30% PPIs removed	8e-35	9e-37	1e-37	2e-34	7e-37	3e-39	2e-42
20% PPIs removed	3e-32	9e-36	4e-34	2e-34	4e-35	1e-38	2e-37
10% PPIs removed	7e-28	9e-30	9e-30	8e-30	2e-30	1e-31	4e-28
**MINT Human**							
50% PPIs removed	4e-16	1e-13	3e-24	1e-24	8e-18	9e-26	7e-22
40% PPIs removed	5e-15	1e-16	6e-22	3e-19	2e-21	2e-23	4e-24
30% PPIs removed	2e-16	1e-19	7e-23	1e-20	2e-20	2e-22	1e-20
20% PPIs removed	3e-17	7e-24	2e-23	2e-19	4e-18	4e-22	2e-20
10% PPIs removed	1e-15	5e-15	3e-15	3e-14	4e-12	2e-17	2e-16
**HuRI Human**							
50% PPIs removed	1e-23	1e-25	9e-30	8e-27	2e-25	3e-28	3e-25
40% PPIs removed	2e-27	2e-27	5e-32	4e-27	5e-28	5e-28	9e-25
30% PPIs removed	1e-23	1e-23	7e-27	1e-23	4e-24	6e-24	4e-22
20% PPIs removed	1e-21	2e-21	2e-22	5e-21	1e-22	2e-23	1e-25
10% PPIs removed	3e-20	2e-18	1e-24	2e-22	9e-20	2e-20	5e-18

Table S4: The same table as Table S1 but for the human datasets. See detailed captions in Table S1.

AUC of PR for rand predictor					
dataset \% of PPIs removed	50%	40%	30%	20%	10%
BioGRID Yeast	7.582e-06	4.726e-06	2.267e-06	1.262e-06	3.027e-07
STRING Yeast	2.494e-05	1.529e-05	8.408e-06	3.908e-06	9.452e-07
MINT Yeast	2.247e-06	1.009e-06	5.536e-07	4.933e-07	0.e+00
BioGRID Human	9.917e-07	8.432e-07	6.524e-07	4.690e-07	2.484e-07
STRING Human	2.805e-06	2.51e-06	2.052e-06	1.497e-06	8.719e-07
MINT Human	3.848e-07	3.310e-07	3.485e-07	2.235e-07	1.273e-07
HuRI	1.226e-06	9.521e-07	8.145e-07	3.698e-07	4.853e-07
Synthetic	6.825e-07	/	/	/	/

Table S5: The rand link predictor's mean PR AUC-values after removing varying numbers of PPIs from the datasets (ten trials for each). The table shows that it is more difficult to pick a real PPIs among the candidate PPIs as the percentage of removed PPIs decreases.

	CN-based	L3-based	CN & L3-based	CRA & L3N'(f_1)
**BioGRID Yeast**				
50% PPIs removed	69%	79 ± 10 %	30 ± 6 %	35%
40% PPIs removed	73%	64 ± 15 %	30 ± 9 %	41%
30% PPIs removed	74%	64 ± 14 %	31 ± 11 %	45%
20% PPIs removed	74%	59 ± 16 %	31 ± 13 %	46%
10% PPIs removed	76%	52 ± 18 %	31 ± 16 %	45%
**STRING Yeast**				
50% PPIs removed	89%	92 ± 2 %	72 ± 6 %	74%
40% PPIs removed	91%	81 ± 15 %	66 ± 15 %	78%
30% PPIs removed	93%	79 ± 15 %	68 ± 16 %	81%
20% PPIs removed	94%	77 ± 17 %	68 ± 18 %	83%
10% PPIs removed	94%	75 ± 16 %	68 ± 18 %	84%
**MINT Yeast**				
50% PPIs removed	43%	72 ± 8 %	32 ± 2 %	34%
40% PPIs removed	74%	65 ± 12 %	30 ± 6 %	35%
30% PPIs removed	61%	64 ± 14 %	39 ± 8 %	52%
20% PPIs removed	30%	66 ± 13 %	45 ± 10 %	57%
10% PPIs removed	75%	67 ± 13 %	49 ± 11 %	63%

Table S6: Overlap ratios of predicted PPIs between different types of link predictors for the yeast datasets across different sample sizes. 'CN-based' and 'CRA & L3N'(f_1)' denotes the overlap ratio of the predicted PPIs between CN and CRA, and between CRA and L3N'(f_1) respectively. For 'L3-based', since there are multiple L3-based predictors (L3, CH2, Sim, L3N'(f_1), and L3N'(f_2)), we calculated the overlap ratio for each pair of predictors. We then took the mean of these ratios as the final value, and also computed the standard deviation. The same applies to 'CN & L3-based' where a CN predictor is compared to a L3-based predictor. Blue color denotes a relatively higher overlap ratio and red a relatively smaller overlap. Ratios are rounded to nearest integers.

	CN-based	L3-based	CN & L3-based	CRA & L3N'(f_1)
**BioGRID Human**				
50% PPIs removed	64%	69 ± 4 %	38 ± 4 %	37%
40% PPIs removed	67%	70 ± 4 %	43 ± 3 %	44%
30% PPIs removed	67%	62 ± 11 %	44 ± 6 %	48%
20% PPIs removed	64%	61 ± 6 %	44 ± 4 %	50%
10% PPIs removed	64%	57 ± 7 %	43 ± 7 %	52%
**STRING Human**				
50% PPIs removed	54%	58 ± 4 %	44 ± 3 %	44%
40% PPIs removed	54%	60 ± 5 %	46 ± 5 %	55%
30% PPIs removed	54%	56 ± 2 %	45 ± 3 %	51%
20% PPIs removed	56%	60 ± 8 %	46 ± 4 %	49%
10% PPIs removed	59%	55 ± 2 %	48 ± 4 %	52%
**MINT Human**				
50% PPIs removed	37%	71 ± 10 %	4 ± 2 %	5%
40% PPIs removed	49%	71 ± 9 %	5 ± 2 %	6%
30% PPIs removed	39%	69 ± 10 %	8 ± 3 %	11%
20% PPIs removed	31%	65 ± 11 %	10 ± 5 %	17%
10% PPIs removed	32%	56 ± 15 %	11 ± 6 %	18%
**HuRI**				
50% PPIs removed	64%	79 ± 7 %	24 ± 3 %	23%
40% PPIs removed	70%	79 ± 7 %	26 ± 3 %	26%
30% PPIs removed	69%	78 ± 7 %	29 ± 3 %	29%
20% PPIs removed	67%	76 ± 7 %	31 ± 4 %	33%
10% PPIs removed	61%	71 ± 9 %	36 ± 6 %	42%

Table S7: Overlap ratios of predicted PPIs between different types of link predictors for the human datasets across different sample sizes. 'CN-based' and 'CRA & L3N'(f_1)' denotes the overlap ratio of the predicted PPIs between CN and CRA, and between CRA and L3N'(f_1) respectively. For 'L3-based', since there are multiple L3-based predictors (L3, CH2, Sim, L3N'(f_1), and L3N'(f_2)), we calculated the overlap ratio for each pair of predictors. We then took the mean of these ratios as the final value, and also computed the standard deviation. The same applies to 'CN & L3-based' where a CN predictor is compared to a L3-based predictor. Blue color denotes a relatively higher overlap ratio and red a relatively smaller overlap. Ratios are rounded to nearest integers.

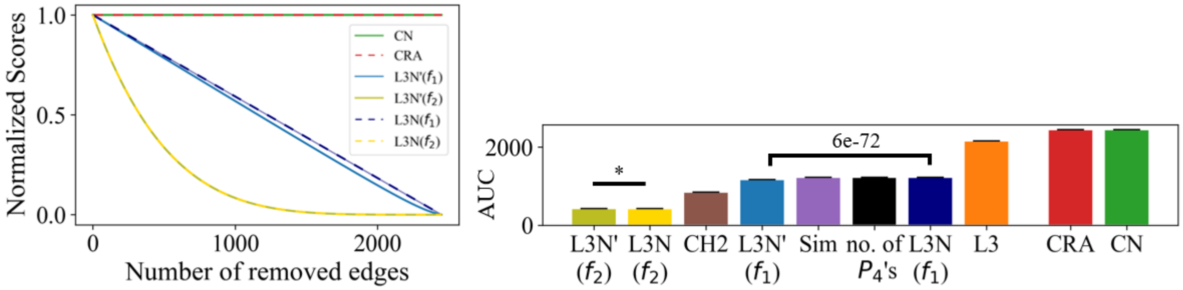

(a) Changes in prediction scores due to the removal of compatible edges in ideal L3 graphs

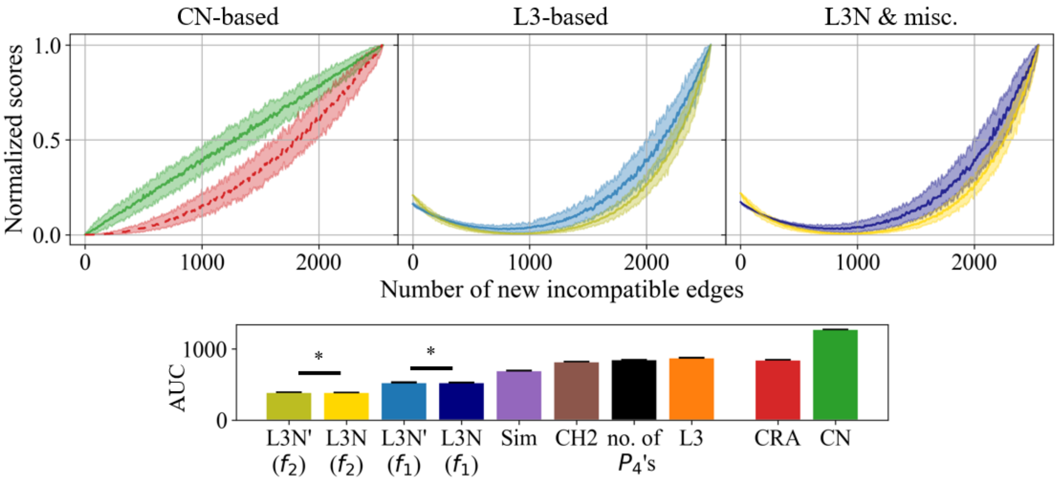

(b) Changes in prediction scores due to the addition of incompatible edges in ideal L3 graphs

Figure S1: Changes in scores for L3N link predictors when an ideal L3 graph is modified by: (a) removing compatible edges; and (b) adding incompatible edges. The shaded regions denote the variance (the minimum and maximum values) among replication experiments, and the solid lines denote the medians. The AUC bar charts correspond to the respective plots (the lower, the better). The statistical bars denote the statistical significance (p-value) between the two corresponding samples using student's t-test. An asterisk denotes $p > 0.05$ and is therefore not statistically significant. In (b), a Savitzky–Golay filter using a polynomial of degree 3 and a window size of 21 was applied to make the curves smoother.

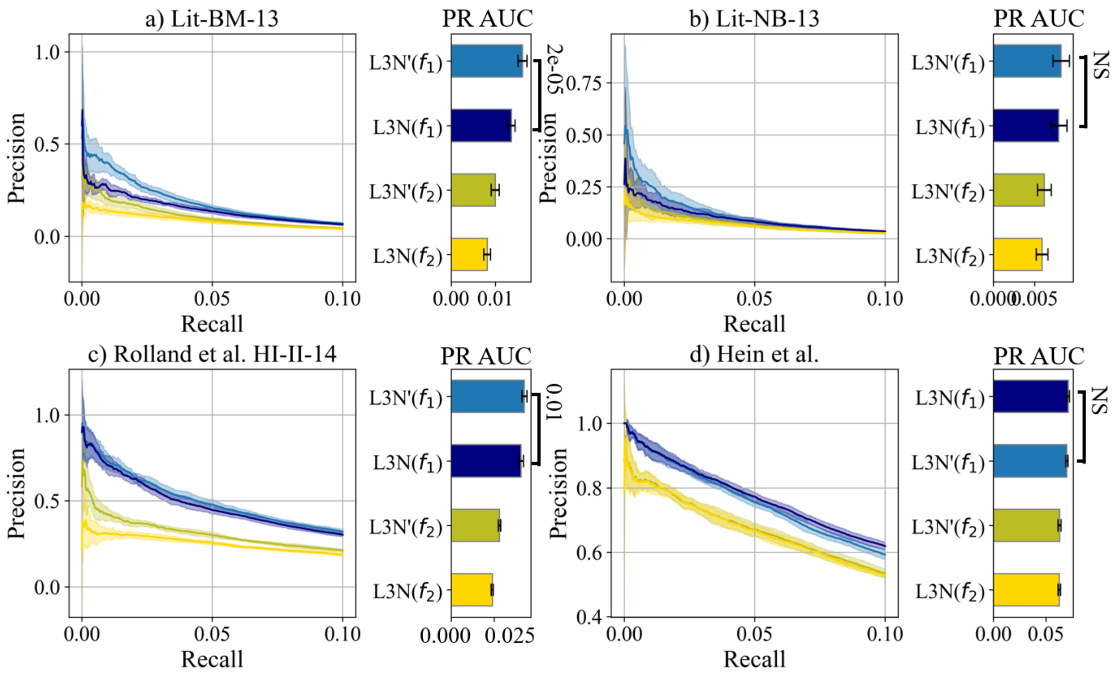

Figure S2: Precision-Recall (PR) curves of all variations of L3N link predictors, computed in the primary datasets used in the study by Kovács *et al.* (2019) under the same methodology (50% of the PPIs removed, computations repeated for 10 times, shaded regions indicate the standard deviations, PR is calculated until 10% of recall has reached). The accompanying bar charts show the predictors' PR AUC-values (the larger, the better).

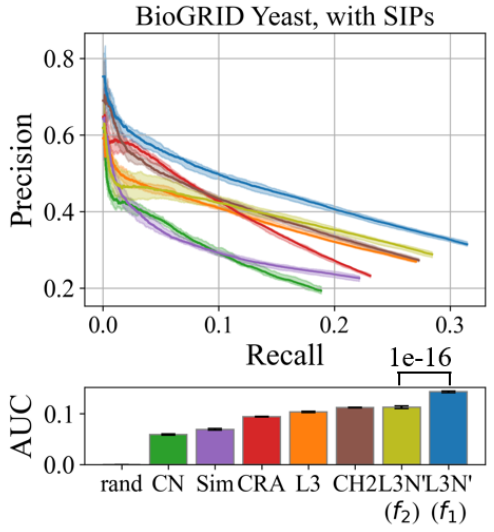

Figure S3: Precision-recall (PR) curves and AUC-values (PR AUCs) of the link predictors computed with 50% of the PPIs removed in the datasets, without self-interacting proteins (SIPs) being removed from the dataset. Only BioGRID Yeast among the others is included since STRING Yeast and MINT Yeast have no SIPs (see Table 1 in the main article). The figure is arranged in the same way as Fig. S2.

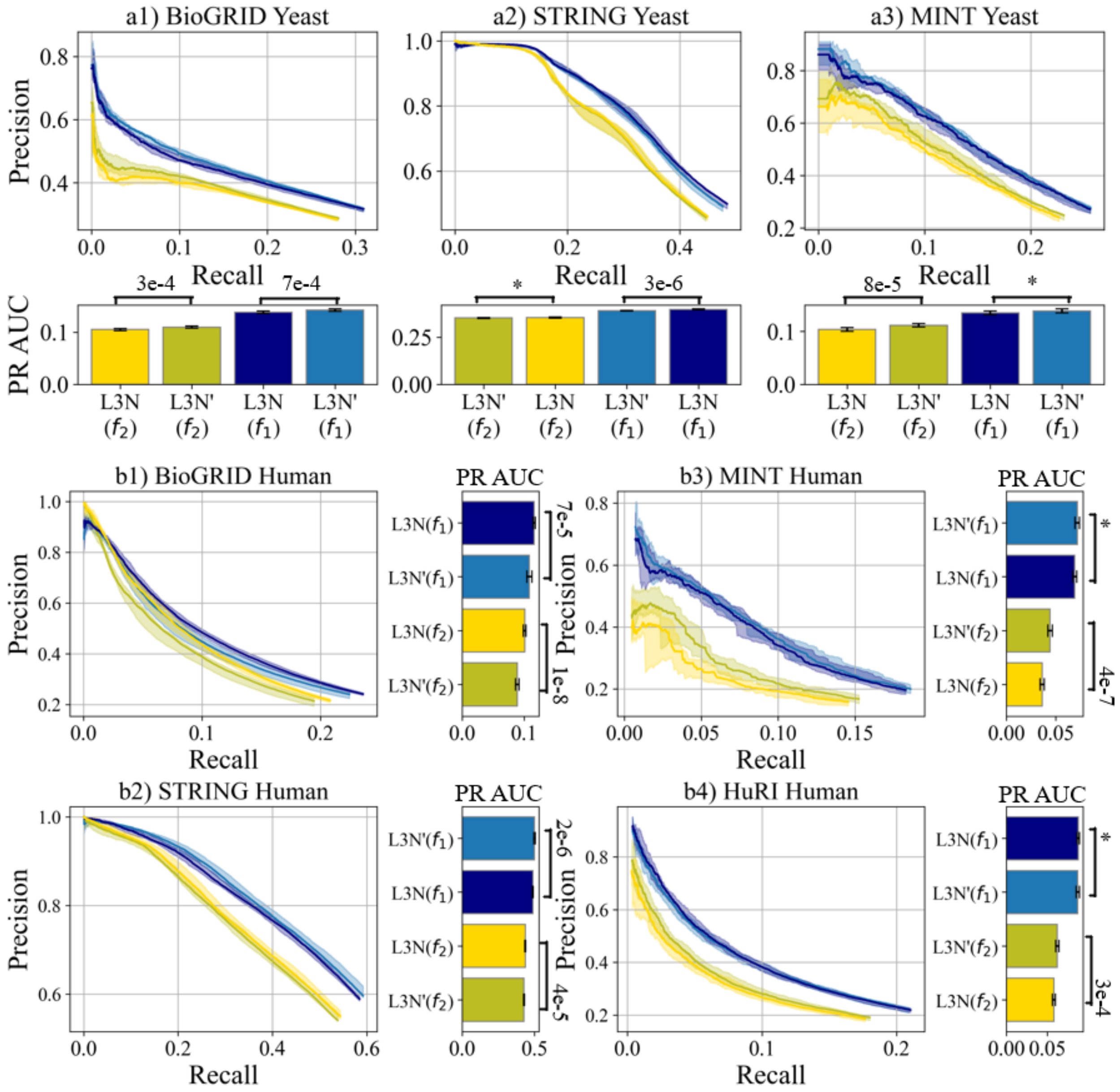

Figure S4: Precision-Recall (PR) curves of the L3N link predictors computed with 50% of the PPIs removed in every dataset. The solid lines show the median values and the shaded regions indicate the variance (the minimum and maximum values). The accompanying bar charts show the predictors' AUC-values (the larger, the better). The statistical bars denote the statistical significance between two samples in terms of the p-value using student's t-test. An asterisk denotes $p > 0.05$ and is therefore not statistically significant.

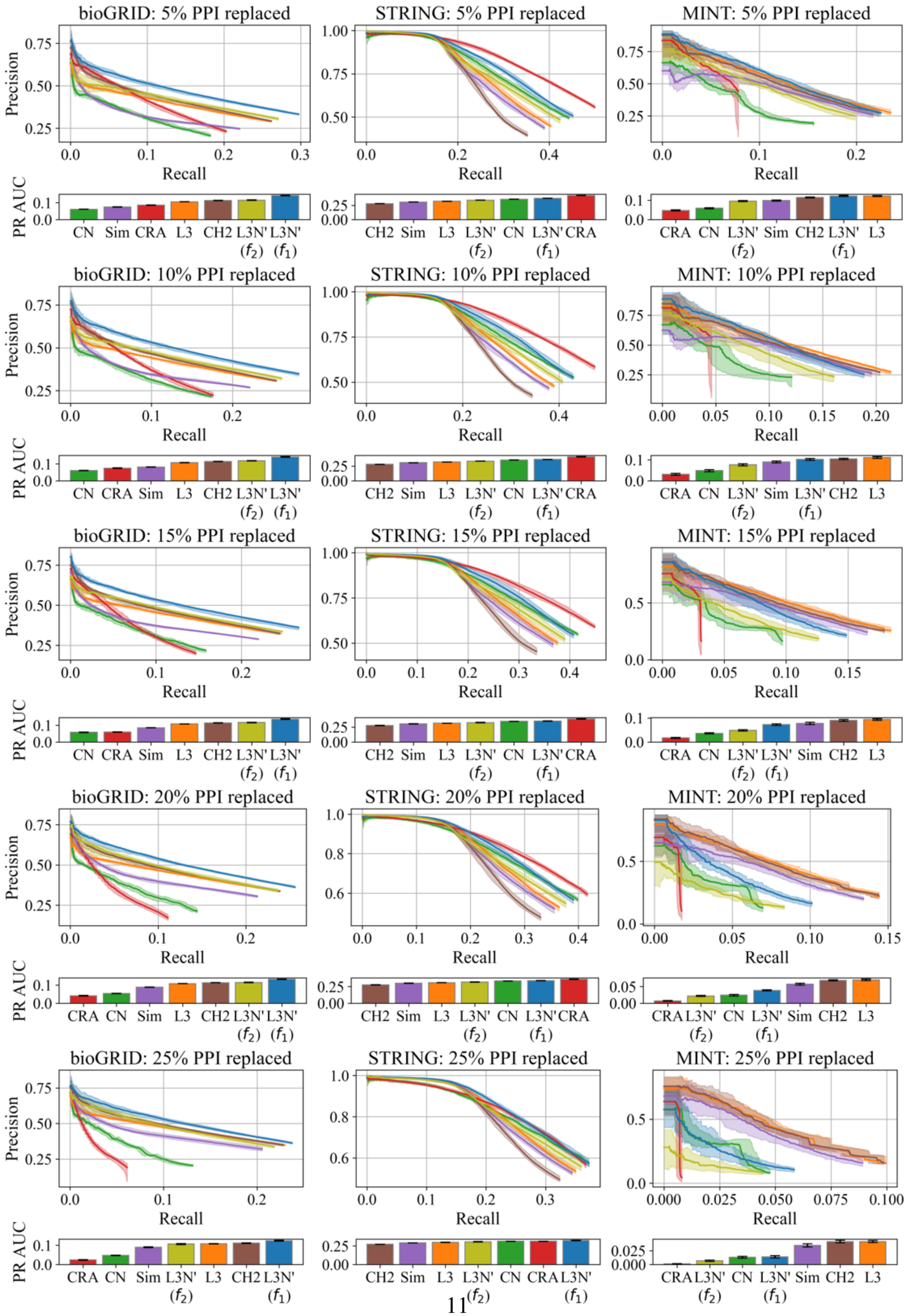

bioGRID: 5% PPI replaced
STRING: 5% PPI replaced
MINT: 5% PPI replaced
bioGRID: 10% PPI replaced
STRING: 10% PPI replaced
MINT: 10% PPI replaced
bioGRID: 15% PPI replaced
STRING: 15% PPI replaced
MINT: 15% PPI replaced
bioGRID: 20% PPI replaced
STRING: 20% PPI replaced
MINT: 20% PPI replaced
bioGRID: 25% PPI replaced
STRING: 25% PPI replaced
MINT: 25% PPI replaced
Precision
Recall
PR AUC
CN Sim CRA L3 CH2 L3N' (f_2) L3N' (f_1)
CH2 Sim L3 L3N' (f_2) CN L3N' (f_1) CRA
CRA CN L3N' (f_2) Sim CH2 L3N' (f_1) L3
CN CRA Sim L3 CH2 L3N' (f_2) L3N' (f_1)
CH2 Sim L3 L3N' (f_2) CN L3N' (f_1) CRA
CRA CN L3N' (f_2) Sim L3N' (f_1) CH2 L3
CN CRA Sim L3 CH2 L3N' (f_2) L3N' (f_1)
CH2 Sim L3 L3N' (f_2) CN L3N' (f_1) CRA
CRA CN L3N' (f_2) L3N' (f_1) Sim CH2 L3
CRA CN Sim L3 CH2 L3N' (f_2) L3N' (f_1)
CH2 Sim L3 L3N' (f_2) CN L3N' (f_1) CRA
CRA L3N' (f_2) CN L3N' (f_1) Sim CH2 L3
CRA CN Sim L3N' (f_2) L3 CH2 L3N' (f_1)
CH2 Sim L3 L3N' (f_2) CN CRA L3N' (f_1)
CRA L3N' (f_2) CN L3N' (f_1) Sim CH2 L3

Figure S5: (cont.) Precision-Recall (PR) curves of the L3N link predictors computed with 50% of the PPIs sampled from the datasets and further processed by replacing either 5%, 10%, 15%, 20%, or 25% of the PPIs by non-PPIs.

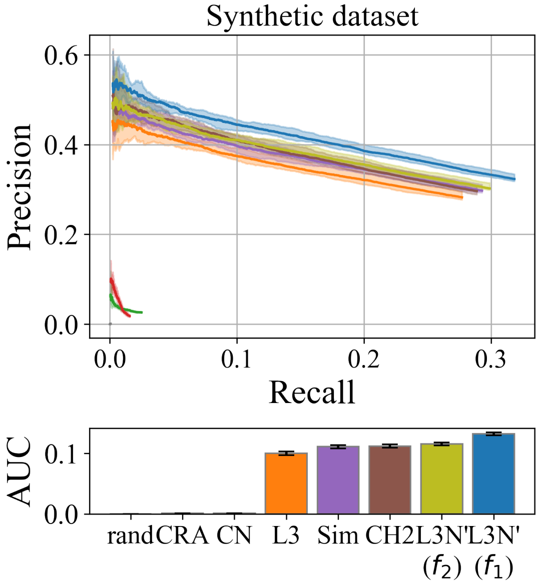

Figure S6: Precision-recall (PR) curves and AUC-values (PR AUCs) of the link predictors computed with 50% of the PPIs removed in the synthetic dataset. As noted in the main article, the method used to generate the dataset gives an advantage to L3-based predictors.

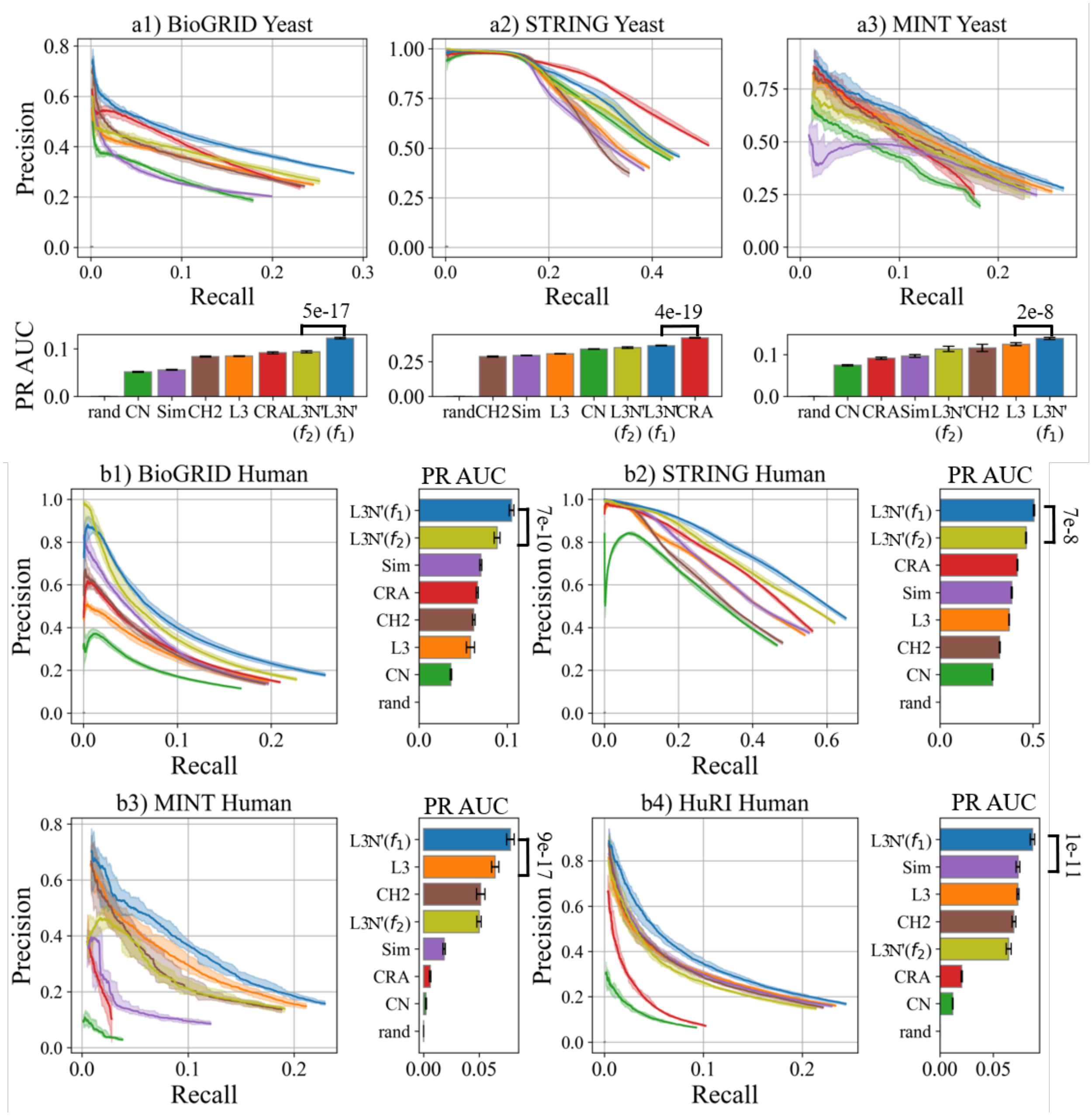

Figure S7: Precision-Recall (PR) curves of the link predictors computed with 40% of the PPIs removed in every dataset. (See the caption of Fig. S4 for explanations.)

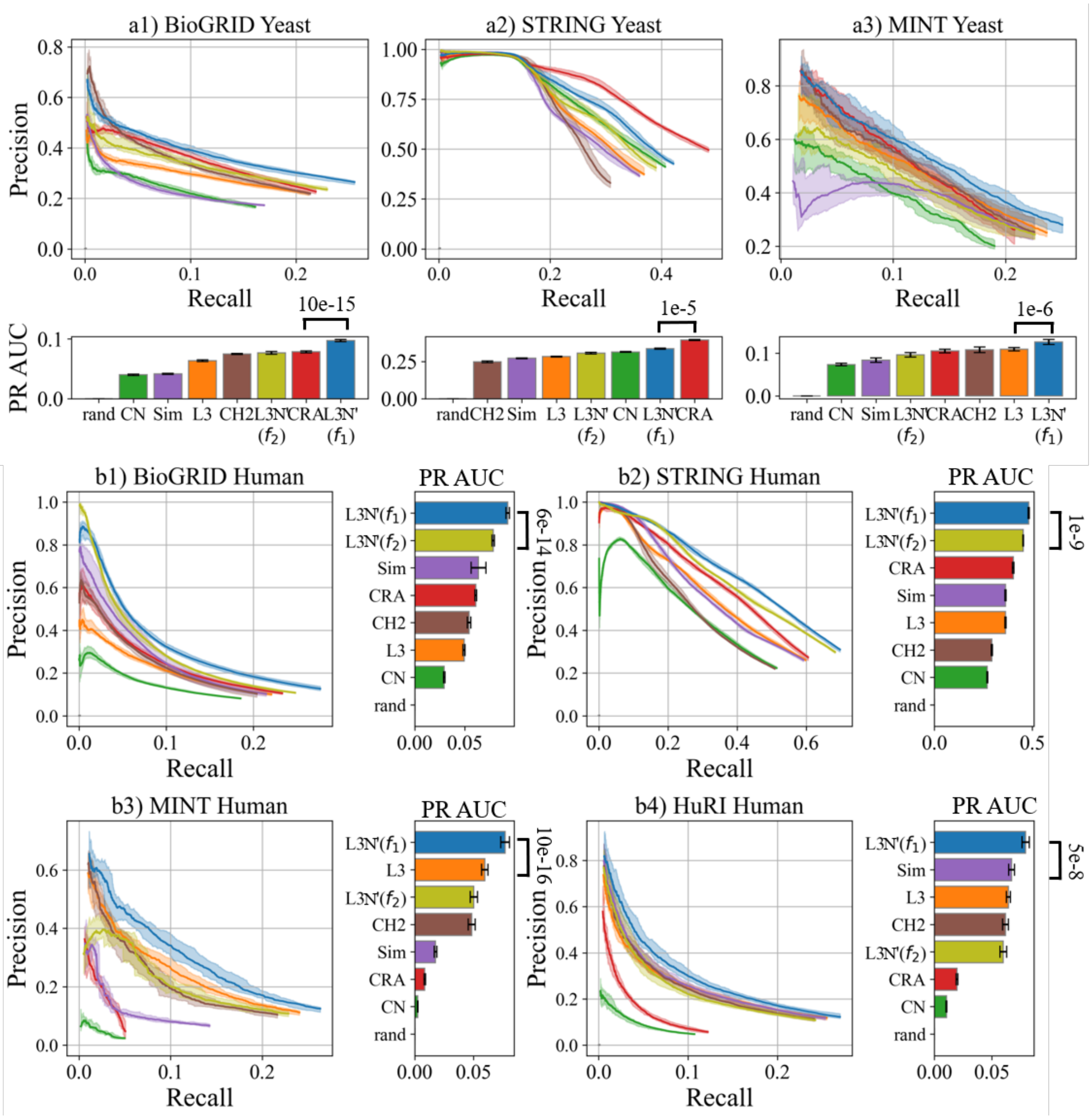

Figure S8: Precision-Recall (PR) curves of the link predictors computed with 30% of the PPIs removed in every dataset. (See the caption of Fig. S4 for explanations.)

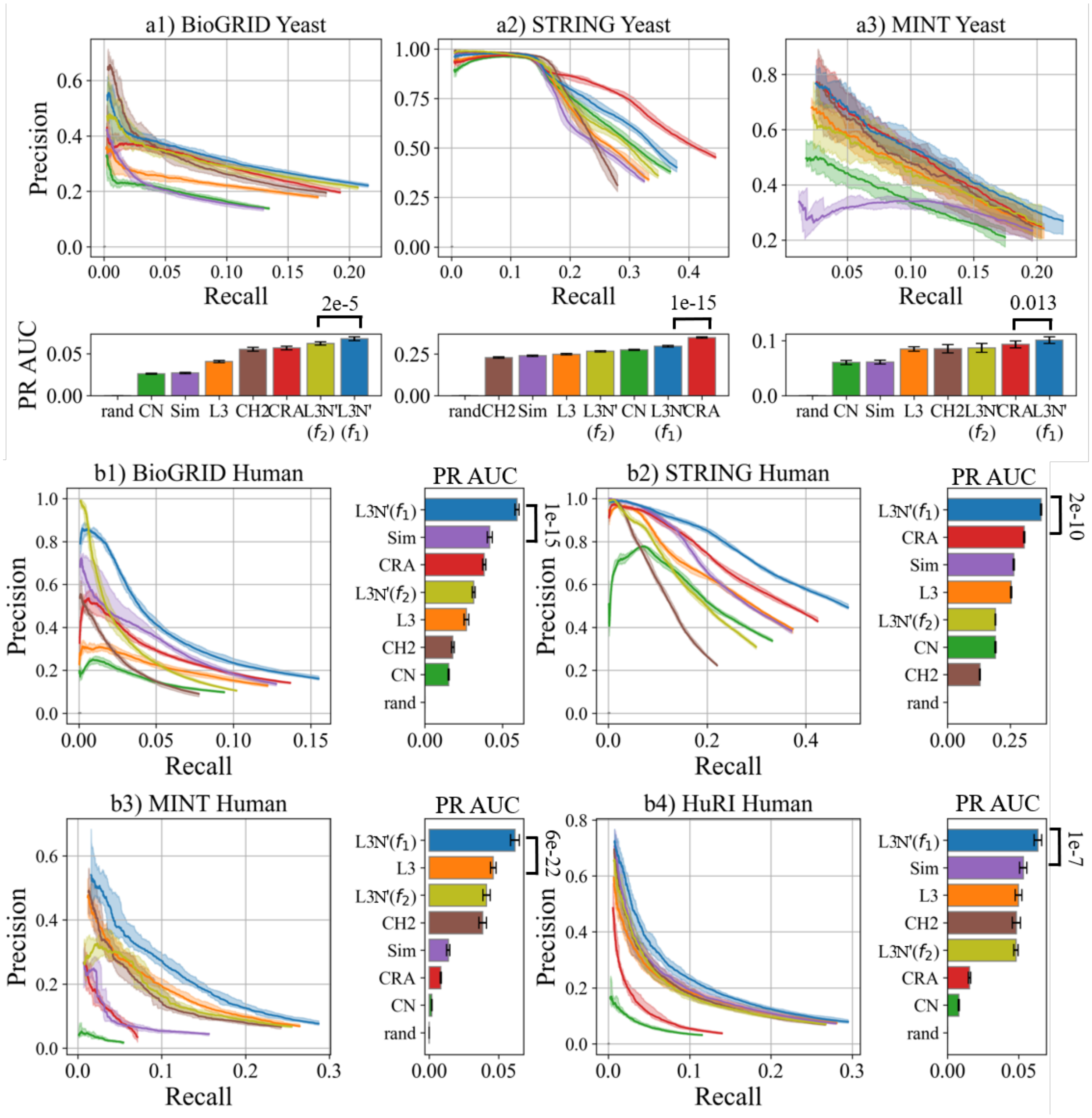

Figure S9: Precision-Recall (PR) curves of the link predictors computed with 20% of the PPIs removed in every dataset. (See the caption of Fig. S4 for explanations.)

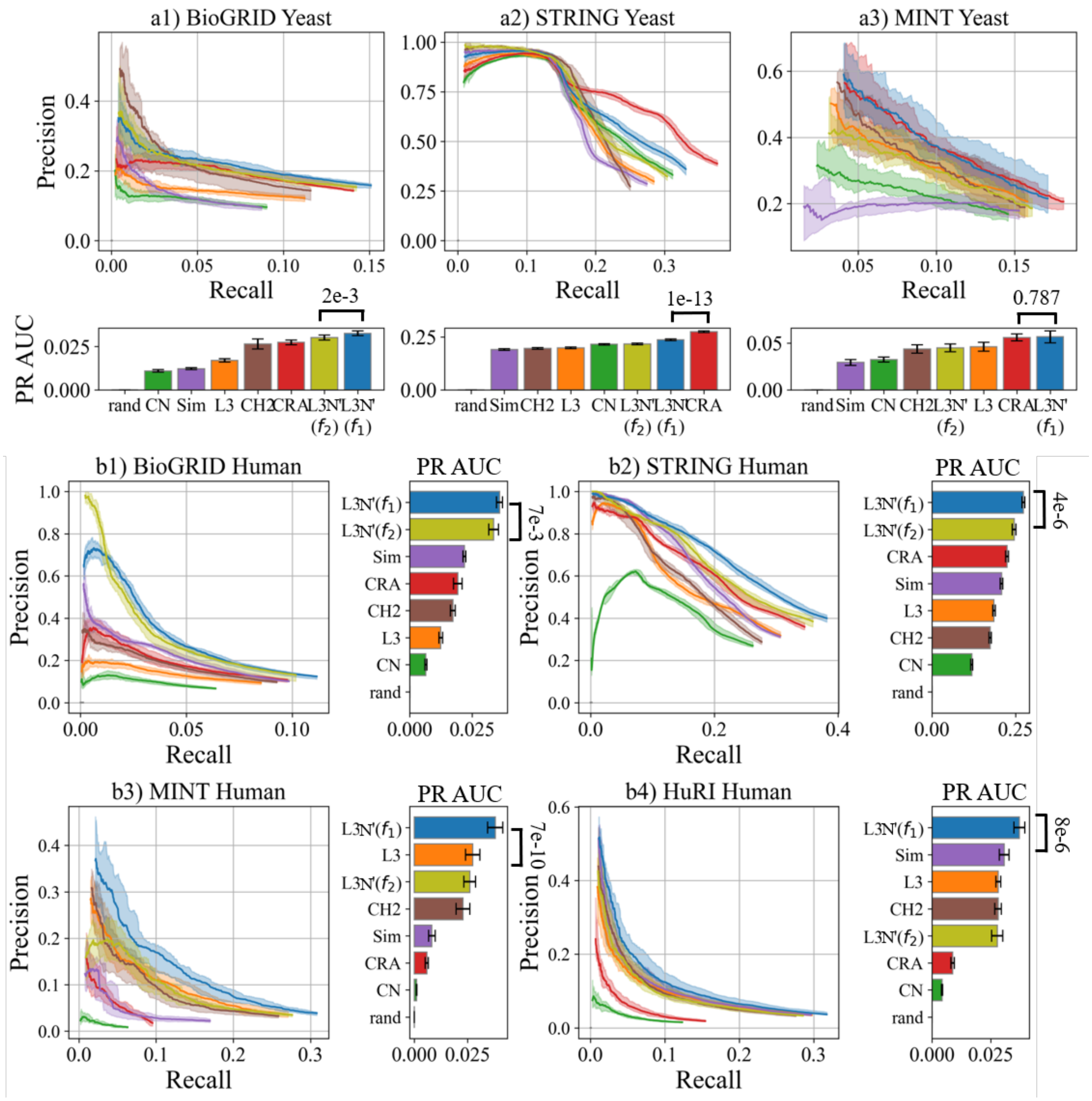

Figure S10: Precision-Recall (PR) curves of the link predictors computed with 10% of the PPIs removed in every dataset. (See the caption of Fig. S4 for explanations.)

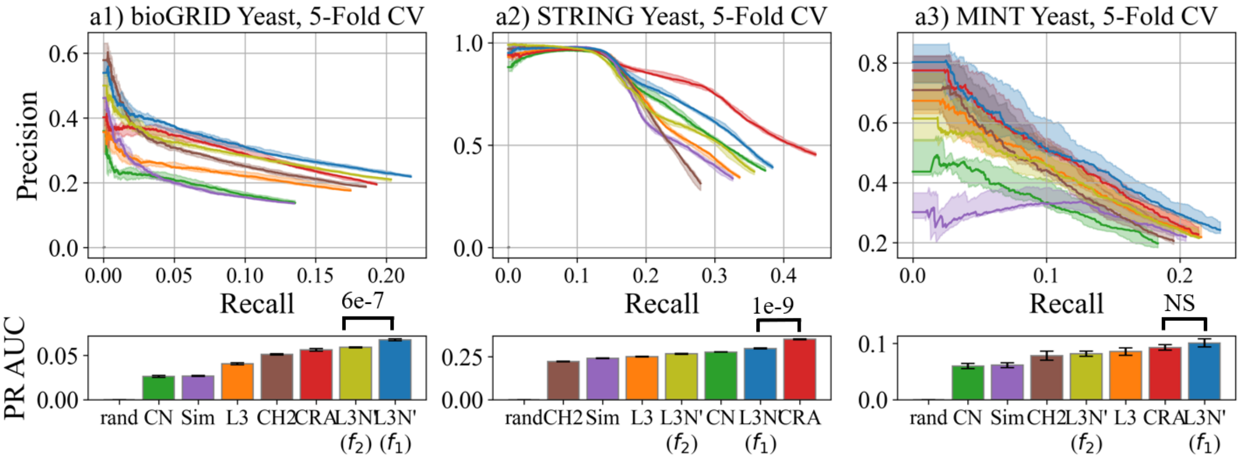

Figure S11: Precision-recall (PR) curves and AUC-values (PR AUCs) of the link predictors computed with the sampled datasets prepared using 5-fold cross validation (5-Fold CV). The figures are arranged the same way as the figures above.

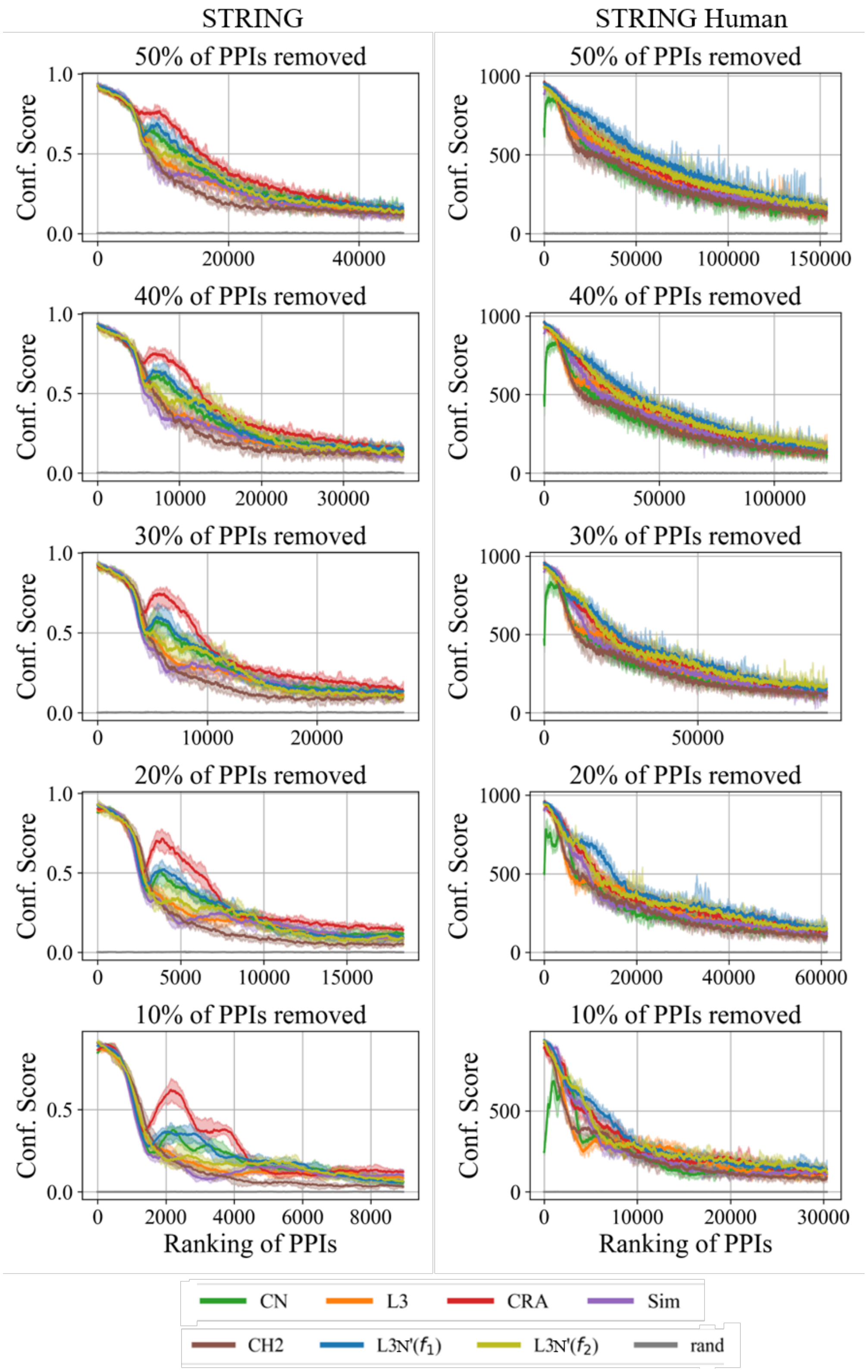

Figure S12: The moving means of the STRING confidence scores across different sample sizes using a window size of 100, with 10 steps forward in each iteration. The shaded regions illustrate the variance (the minimum and maximum values) in STRING confidence scores.

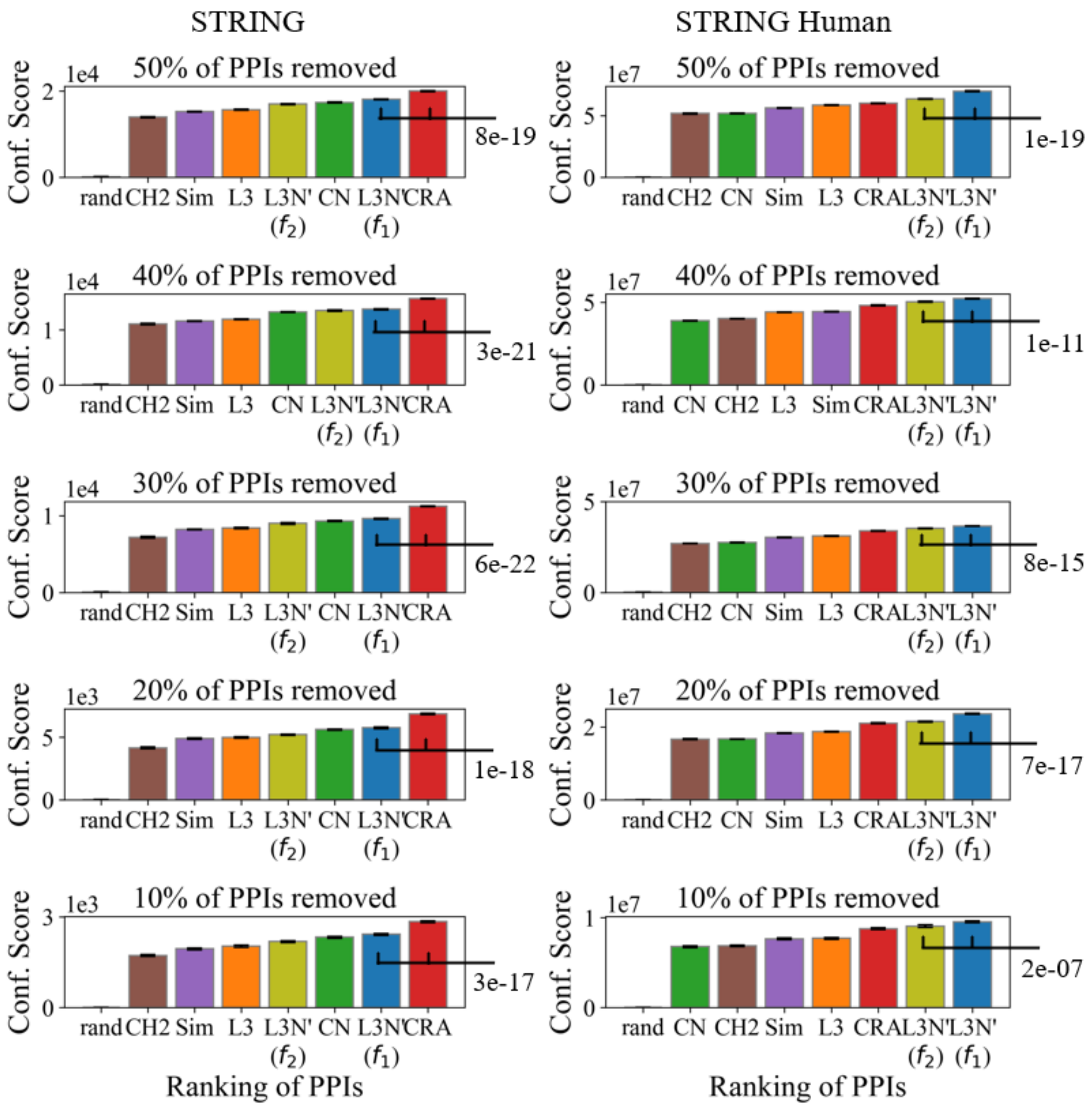

Figure S13: AUCs corresponding to the curves in Fig. S12. The predictors are sorted from left to right (worst to best) according to their AUC-values. Statistical bars denote the statistical significance between two samples in terms of p-value using student's t-test.

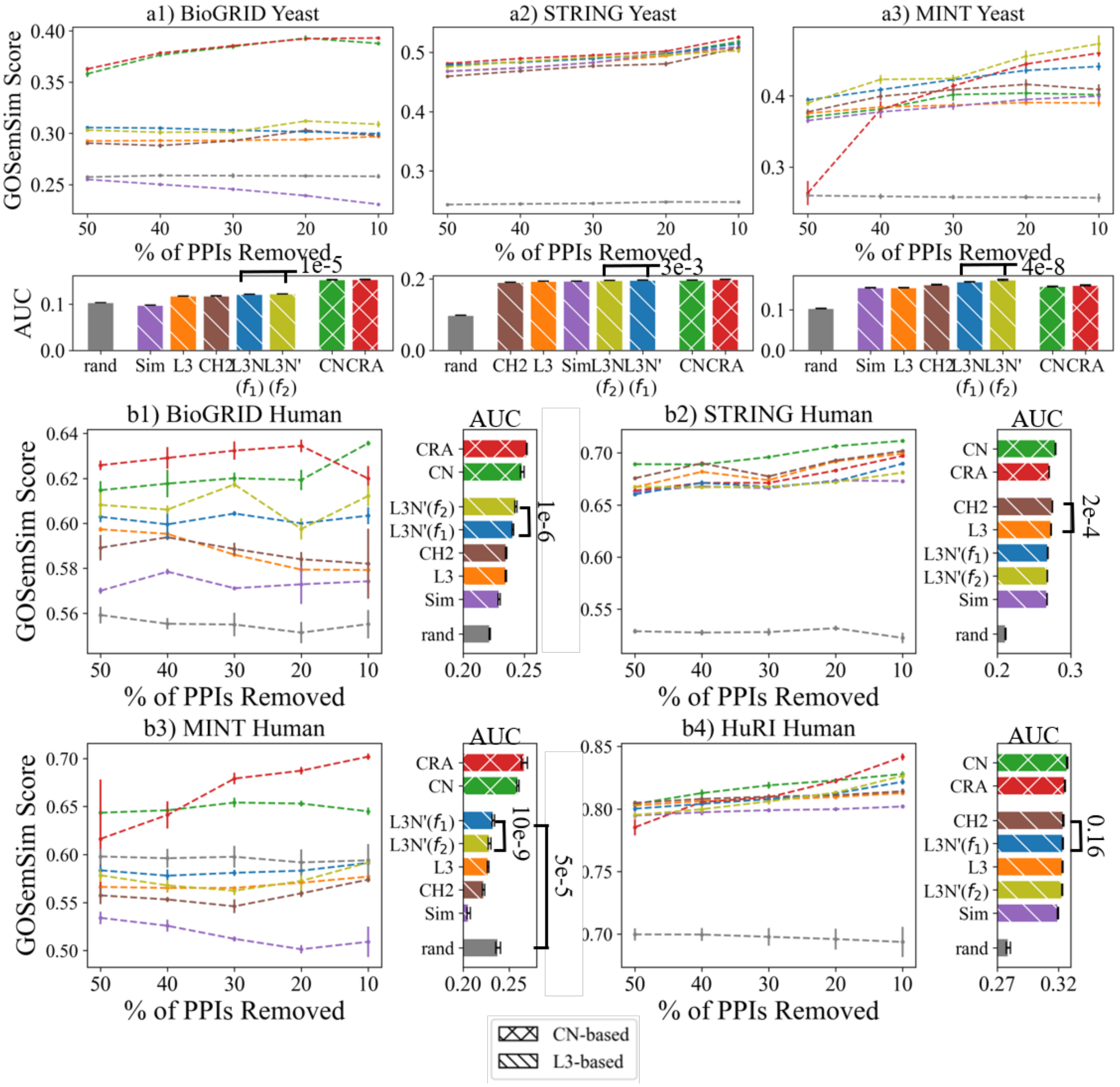

Figure S14: Illustrating how the mean GOSemSim score of the link predictors changes as the percentage of removed PPIs decreases. The dotted curves are interpolations of the data points (50%, 40%, 30%, 20%, 10%) and the vertical bars on the data points indicate the variance (the minimum and maximum values). The gray curve (rand) is the negative control. The bar charts show the AUCs of the GOSemSim scores and the statistical bars give the statistical significance between two samples in terms of the p-value using student's t-test.
